# Density-dependence and environmental variability have stage-specific influences on European grayling growth

**DOI:** 10.1007/s00442-022-05163-2

**Published:** 2022-05-04

**Authors:** Jessica E. Marsh, Richard J. Cove, J. Robert Britton, Robert G. Wellard, Tea Bašić, Stephen D. Gregory

**Affiliations:** 1 Salmon and Trout Research Centre, Game and Wildlife Conservation Trust, The River Laboratory, Wareham, Dorset UK; 2 Department of Life and Environmental Sciences, Faculty of Science and Technology, Bournemouth University, Poole, Dorset UK; 3 Centre for Environment, Fisheries and Aquaculture Science (Cefas), Weymouth, Dorset UK; 4 Natural Resources Wales/Cyfoeth Naturiol Cymru, Buckley, Flintshire UK; 5 The Piscatorial Society, Wiltshire, UK; 6 Salmon and Freshwater Team, Centre for Environment, Fisheries and Aquaculture Science (Cefas), Lowestoft, Suffolk UK

**Keywords:** Fisheries management, Inter-specific and intra-specific competition, Lowland river, Von Bertalanffy

## Abstract

Fish somatic growth is indeterminate and can be influenced by a range of abiotic and biotic variables. With climate change forecast to increase the frequency of warming and unusual discharge events, it is thus important to understand how these variables currently influence somatic growth and how that might differ for specific age-classes and/ or life stages. Here, we used a 17-year dataset from a chalk stream in southern England to identify the abiotic and biotic influences on the growth of juvenile, sub-adult and adult life stages of European grayling (*Thymallus thymallus*), a cold-water riverine salmonid. The results revealed that interannual variations in grayling growth were well described by annual- and site-specific abiotic and biotic explanatory variables. We found divergent responses between life stages to increased temperature and unusual discharge during the main growth period with, for example, elevated temperatures related to increased juvenile growth but reduced sub-adult growth, and high discharge events related to increased sub-adult growth yet reduced juvenile growth. Conversely, stage-specific grayling abundance negatively influenced growth at each life stage, though only juvenile growth was impacted by the abundance of a competitor species, brown trout (*Salmo trutta*). These results emphasise the merits of testing a wide range of environmental and biological explanatory variables on fish growth, and across life stages. They also reveal the importance of maintaining high habitat heterogeneity in rivers to ensure all life stages can reduce their competitive interactions and have access to adequate flow and thermal refugia during periods of elevated environmental stress.

**Supplementary Information:**

The online version contains supplementary material available at 10.1007/s00442-022-05163-2.

## Introduction

Somatic growth in fish is indeterminate and influenced by a range of abiotic and biotic variables (Charnov and Berrigan, 1991). Individual growth strongly influences ultimate body size and reproductive fitness (Barneche et al. 2018; Tréhin et al. 2021), and is thus an important aspect of population dynamics (Plard et al. 2015). Abiotic and biotic drivers of fish growth, and/or the direction and magnitude of their effects, are likely to vary between life stages as habitat and resource requirements, physiological tolerances and sexual maturity, shift during ontogeny (Lange et al. 2018; Stoffels et al. 2020). For example, the relationship between energy acquired and somatic growth changes profoundly at maturation, whereafter much of the energy acquired is allocated to reproduction, with a concomitant decline in somatic growth (Lester et al. 2004). Yet, although age- or stage-specific growth responses to their physical and biological environment are important for decoupling their effects across multiple life stages, studies incorporating age- or stage-specific analyses remain rare, especially in freshwater systems.

Studies on stage-specific growth in riverine fish often focus on the influence that abiotic variables, especially discharge and temperature, have on different life stages. For example, Stoffels et al. (2020) detected a negative influence of extreme low annual discharge on the growth of all life stages of Murray cod (*Maccullochella peelii*) and, although flood events benefitted adult growth, they resulted in sub-optimal conditions for juvenile growth. Furthermore, the temperatures required for the fastest growth rates increased with age (Stoffels et al. 2020). Interactions between these abiotic variables can also influence the strength and direction of stage-specific growth responses. For example, in brook trout (*Salvelinus fontinalis*), elevated flows resulted in faster growth rates of older (1 +) individuals, whereas an interaction of elevated flows and higher water temperature was required to increase juvenile growth rates (Letcher et al. 2015).

Unusual river discharge and temperature conditions are expected to occur more frequently as climate change increasingly disrupts hydrological and thermal regimes (Reid et al. 2018; Gudmundsson et al. 2021). Therefore, it is imperative to identify how they have influenced growth in wild fish populations over extended periods, particularly as they tend to act in a density-independent manner (Beardsley and Britton, 2012a, b). However, to fully understand their influences on fish growth, a range of other variables need to be considered, especially biotic variables that are more likely to influence growth in a density-dependent manner through their influence on intra- and inter-specific competition (Ward et al. 2006; Amundsen et al. 2007). For example, declines in prey availability and accessibility caused a population crash of rainbow trout (*Oncorhynchus mykiss*), partly due to increased competition, reduced growth rates and body condition (Korman et al. 2021). In salmonid fishes generally, the influence of density-dependent processes on growth can be strong in both young-of-year and adult life stages (Grossman and Simon 2020). Nevertheless, quantifying the relative impact of abiotic and biotic variables on stage-specific growth can be complex, but is necessary to understand how populations respond to long-term changes in their environment, including changes in the abundance of their competitor and prey populations.

In this study we aim to identify stage-specific influences on the growth of juvenile, sub-adult and adult life stages of European grayling (*Thymallus thymallus* hereafter, grayling), a cold-water riverine salmonid that are native to much of northern Europe (Ibbotson et al. 2001). Grayling populations are relatively understudied compared to many other salmonid species (Bašić et al. 2018), resulting in a paucity of knowledge on the relative importance of abiotic and biotic variables to their populations. However, we consider grayling a strong model fish species for testing stage-specific influences on growth as, unlike many riverine salmonids, they have a potamodromous life-history whereby all life stages inhabit the freshwater, and so their populations will be more sensitive to altered freshwater conditions than other salmonid species, such as Atlantic salmon (*Salmo salar*) and brown trout (*S. trutta*) (Ibbotson et al. 2001). They have also been described as a suitable indicator species for future impacts of environmental change on other taxa (Bašić et al. 2018; Huml et al. 2020), as they are sensitive to changes in water quality and elevated temperature, particularly relative to other salmonid species (Ibbotson et al. 2001; Uiblein et al. 2001; Jonsson and Jonsson, 2009; Huml et al. 2020).

This study is located on the River Wylye, a chalk stream in southern England, where the grayling population has been consistently monitored at six sites since 1996 (Marsh et al. 2021). As this population is situated towards the species’ southern range limit, their population responses to changes in the freshwater environment potentially act as an early warning for salmonid populations at higher latitudes. Southern England chalk streams are threatened by climate change, confounded by human activities (CaBA 2021), and specific studies have shown increases in their average seasonal temperatures since 1989 (Durance and Ormerod 2009). In the River Wylye, recent environmental changes have been detected (e.g. discharge regimes becoming asynchronous with seasonal change), with consequent abundance declines in all grayling age-classes (0 + to 5 + years), while the sympatric brown trout population has remained stable (Marsh et al. 2021). There is a growing literature suggesting that these and other environmental changes are related to body size decreases in many species, from plants to fish (Sheridan and Bickford 2011), including salmonids (e.g., Gregory et al. 2017). Consequently, we test for stage-specific influences of temperature, discharge, prey resources, in-river habitat, and conspecific and heterospecific abundance variables to discern how long-term variability in environmental conditions and intra- and inter-specific competitor abundance influenced interannual differences in stage-specific grayling growth. We specifically developed a range of a priori hypotheses from existing knowledge to test the influence of these variables on grayling growth at three life stages: juveniles (age 0 +), sub-adults (age 1 +) and adults (ages 2 + to 5 +) (Table 1).

**Table 1:** Abiotic and biotic explanatory variables and hypothesised direction of influence on life stage-specific growth and length at age

Variable	Description	Hypothesis	References	Direction/ life stage
*Temperature*				
Mean temperature (Spring–Autumn)	Mean air temperature between 1st April and 30th September as a proxy for water temperature	Years with higher average temperatures promote growth potential, resulting in larger length at age	Deegan et al. (1999) Mallet et al. (1999) Luecke and MacKinnon (2008)	+ Juvenile, sub-adult and adult
Mean temperature (Autumn–Winter)	Mean air temperature during autumn–winter period prior to sampling (between 1st October and 31st March) as a proxy for water temperature	As above	As above	+ Sub-adult and adult
High temperature (Spring–Autumn)	Number of days between 1st April – 30th September where air temperatures exceeded 20 °C (at > 20 °C air temps, mean water temperature in Wylye = 17.5 °C)	Water temperatures exceeding 17 °C negatively affect grayling growth. Years with more days of high temperatures will result in reduced growth potential and smaller length at age	Hobbie et al. (1999) Mallet et al. (1999)	– Juvenile, sub-adult and adult
*Flow*				
Low flow (Spring–Autumn)	Number of days between 1st April – 30th September where mean daily discharge is equal to or less than Q90 (the 10th percentile discharge in the period 2003–2019)	Low discharge can reduce habitat area and invertebrate drift, resulting in less available food resource and lower growth. Years with more days of low discharge will result in smaller length at age	Hobbie et al. (1999)	– Juvenile, sub-adult and adult
High flow (Spring – Autumn)	Number of days between 1st April – 30th September where mean daily discharge is equal to or greater than Q10 (the 90th percentile discharge in the period 2003–2019)	In high discharge, smaller grayling might be unable to maintain swimming and speeds necessary to access drift prey, reducing energy intake and growth potential. Years with more days of high discharge will result in smaller length at age	Deegan et al. (1999)	– Juvenile and sub-adult
*Habitat*				
Macrophyte cover	Mean macrophyte cover during summer	Macrophyte beds reduce access to the benthos and benthic prey resources, reducing energy intake and growth potential. Years of high macrophyte cover will relate to smaller length at age	Ibbotson (1993)	– Juvenile and sub-adult
Macroinvertebrate biomass (Spring–Autumn)	Mean macroinvertebrate biomass index calculated from spring and autumn samples	Energy intake is one of the main parameters governing fish growth. Grayling growth will be higher in years of high food availability, i.e. greater macroinvertebrate biomass, resulting in larger length at age	Elliott (1994) Deegan et al. (1997)	+ Juvenile, sub-adult and adult
*Competitors*				
Conspecific abundance at time of survey	Site-specific estimated abundance of grayling life stages at time of sampling in autumn	High population densities thought to reduce individual grayling growth rates, possibly through antagonistic interactions and reduced food availability. Years with high competitor abundance will relate to smaller length at age	Woolland and Jones (1975) Deegan et al. (1997) Hagelin and Bergman, (2021)	– Juvenile, sub-adult and adult
Heterospecific abundance at time of survey	Site-specific estimated abundance of small (< 150 mm) and large and older (> 150 mm) brown trout at time of sampling in autumn	As above	Hagelin and Bergman (2021)	– Juvenile (small trout) – Sub-adult and adult (large trout)

## Materials and methods

### Fish sampling

**Fig. 1:**
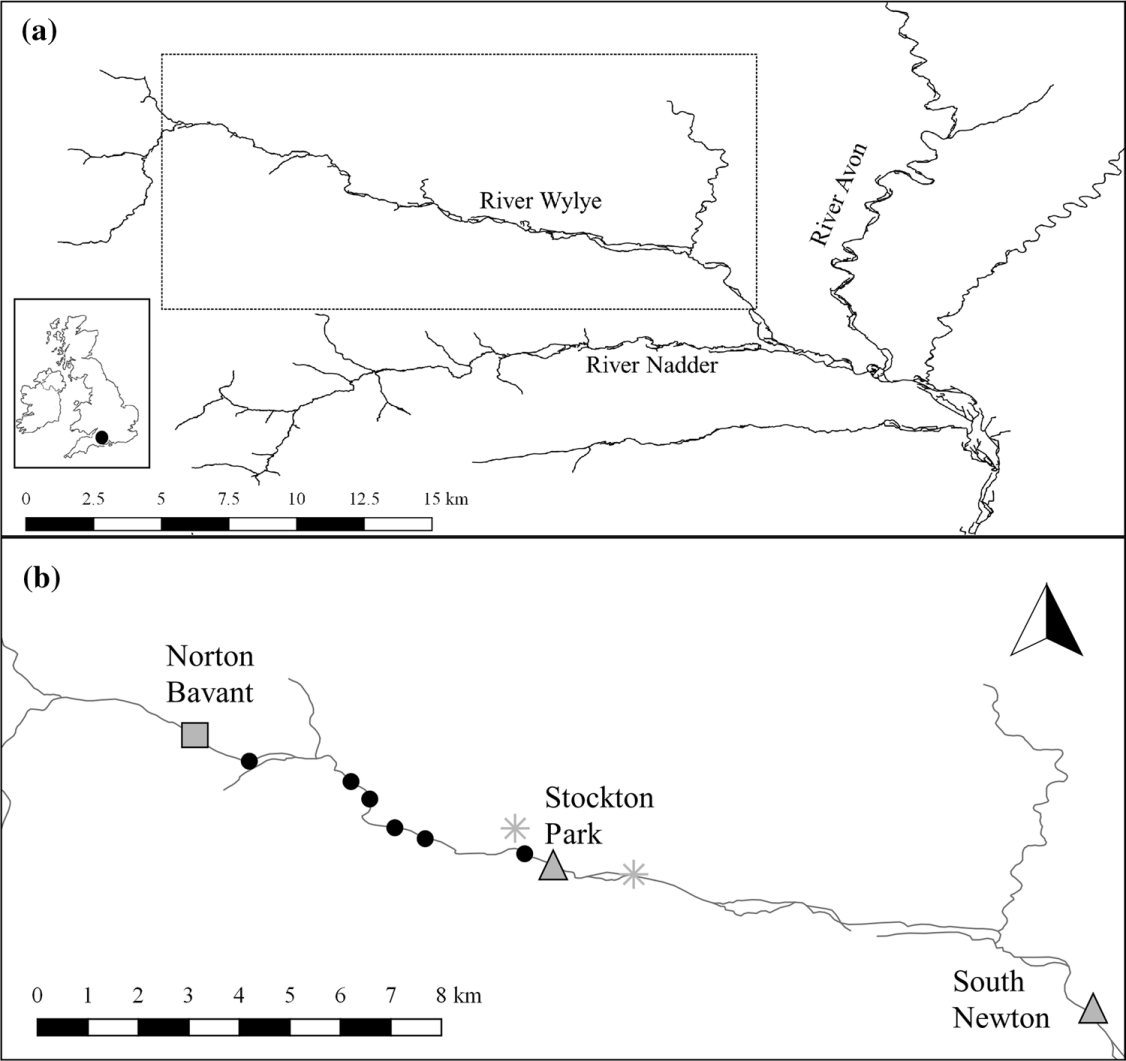
Location of **a** the study area (dashed box) on the River Wylye in the River Avon (Hampshire) catchment and UK (inset map) and **b** long-term fishing sites (black circles) and abiotic and biotic data sampling locations within the study area. Grey symbols show locations of macroinvertebrate sampling at Norton Bavant (square), flow gauging stations at Stockton Park and South Newton (triangles), and the upstream and downstream limits of the macrophyte survey (asterisks)

We used 17 years (2003–2019) of grayling and brown trout population census data collected by the Wylye Grayling and Trout Survey on the River Wylye, a predominately groundwater-fed chalk stream and tributary of the Hampshire Avon, UK (Fig. 1). Grayling and trout were captured in autumn (late-September to early–October) each year using either single pass electrofishing (2003–2008), or multiple (*k* = 3) pass depletion electrofishing (2009–2019) at each site. During fishing, sites of 200 m length (8.4 ± 1.1 m mean width) were closed using stop nets at the downstream and upstream site limits. Captured grayling and trout were removed after each pass and counted. Fish were lightly anaesthetised (2-phenoxyethanol; 0.2 ml/L), identified to species and measured for fork length (nearest mm). A scale sample was taken from each grayling (three to five scales per fish), from between the dorsal fin and adipose fins and above the lateral line, for age determination (Horká et al. 2010). Following their recovery to normal behaviour, all fish were released back alive into the river section where they were captured. All fish processing was carried out by licenced personnel under a UK Home Office A(SP)A project licence (PPL 30/3277). The grayling ages were determined by an experienced scale reader on a projecting microscope (× 20 to × 30 magnification). Grayling age at sampling was verified as one of eight age-classes (0 + to 7 +), although we did not attempt to estimate growth for age 6 + and 7 + grayling in this study owing to too few individuals caught (Fig. S1). Trout were classified as juveniles (fork length ≤ 150 mm) or older, large trout (fork length > 150 mm) based on length-frequency histograms (Fig. S2).

### Abiotic and biotic explanatory variables

**Fig. 2:**
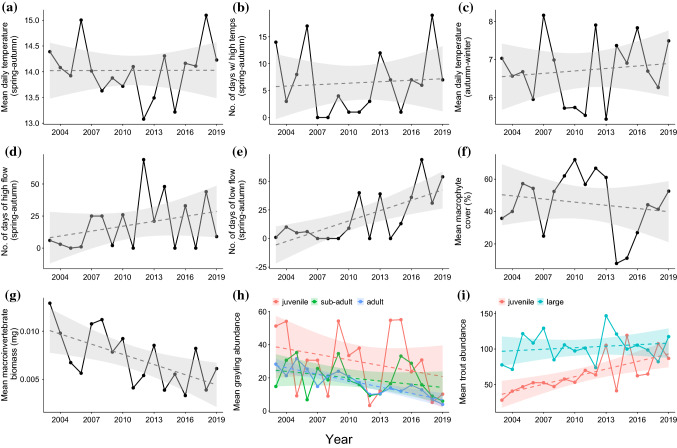
Abiotic and biotic explanatory variables hypothesised to influence European grayling (*Thymallus thymallus*) growth and expected length at age, calculated for *n* = 16 years (and life stage for grayling and brown trout (*Salmo trutta*) abundance). Grayling and trout abundance are shown averaged across site for simplicity but were included in the growth model as year and site-specific variables. The dashed lines indicate the 16-year linear trend with Year, with uncertainty represented as standard error bands

A priori hypotheses were used to test the influence of abiotic and biotic explanatory variables on grayling growth at three life stages (Table 1). Most of the explanatory variables were calculated for the main growing period, defined as April to September (hereafter spring–autumn) based on observations that grayling growth in the River Dee (North Wales) was highest during this period (Woolland and Jones 1975). We also, however, considered temperature influences outside of this period (October to March, hereafter autumn–winter) as chalk streams are groundwater-fed, and as such have relatively stable annual temperatures compared to rain-fed rivers (Berrie 1992), and this promotes an extended period of feeding and growth. We included abiotic explanatory variables to test for the influence of average and unusual temperatures and river discharge on grayling growth (Table 1, Fig. 2a–e). To calculate mean temperature during both growing periods, we used local air temperature estimates from the Europe-wide E-OBS gridded dataset (E-OBS v22.0e; Cornes et al. 2018). We used air temperature as a proxy of water temperature because we lacked consistent water temperature data covering the whole study period and there was a strong relationship between daily mean air and water temperatures in years of available data (Fig. S3; Marsh et al. 2021). For variables representing unusual discharge events, we calculated low and high flow using river discharge (as daily means, m^3^s^−1^) measured at the Stockton Park gauging station from the National River Flow Archive (https://nrfa.ceh.ac.uk; Fig. 1). Missing data were imputed using discharge data from the nearby gauging station at South Newton (Marsh et al. 2021).

We included biotic explanatory variables to characterise foraging habitat and competitor abundance and test for these influences on grayling growth (Table 1, Fig. 2f–i). As an explanatory variable representing potential prey resources, we used macroinvertebrate biomass as calculated in Marsh et al. (2021) from spring and autumn macroinvertebrate abundance estimates sampled at Norton Bavant in a separate monitoring programme (Fig. 1; Environment Agency/Wessex Water). As an explanatory variable representing in-river macrophyte cover, we used percentage cover of macrophytes, predominately *Ranunculus* spp., estimated from 100 m long bankside surveys carried out each summer (late July/early August) at 20 locations at the downstream end of the fishing sites in a separate monitoring programme (Fig. 1; Environment Agency/Wessex Water). Explanatory variables representing the influence of grayling and trout abundance (as a proxy of the strength of intra- and inter-specific competition) on grayling growth were estimated as the abundance of each life stage of grayling (juvenile/sub-adult/adult) and trout (juvenile/older and larger) at the time of the autumn sampling in each site and year of the study. To estimate their abundances, we used an *N*-mixture model where the observation model was represented by a *k*-pass depletion survey, which accounted for imperfect sampling (Wyatt 2002). Abundance was estimated separately for grayling and trout as

$$N_{y,s,a} \sim {\text{Poisson}}\left( {\lambda_{y,s,a} } \right)$$

$$log\left( {\lambda_{y,s,a} } \right) = \alpha_{y,s,a} ,$$

where $N_{y,s,a}$ is the abundance of fish and $\lambda_{y,s,a}$ is the mean expected number of fish in year *y*, site *s* and age-class *a*. The observation model was represented by a sequential series of binomial equations emulating the depletion survey in each *y* (year notation omitted for brevity) given by

$$\begin{array}{*{20}l} {C_{s,a,1} \sim {\text{binomial}}\left( {p_{g} ,N_{s,a} } \right)} \hfill \\ {C_{s,a,2} \sim {\text{binomial}}\left( {p_{g} ,\left( {N_{s,a} - C_{s,a,1} } \right)} \right)} \hfill \\ {C_{s,a,3} \sim {\text{binomial}}\left( {p_{g} ,\left( {N_{s,a} - C_{s,a,1} - C_{s,a,2} } \right)} \right),} \hfill \\ \end{array}$$

where ${C}_{s,a,1:3}$ are the catches of fish of age-class *a* in site *s* in each pass, and ${p}_{g}$ is the individual capture probability that was estimated separately for age 0 + (*g* = 1) and ages 1 + to 5 + (*g* = 2) grayling following Marsh et al. (2021) and estimated for all trout ages together (*g* = 1 only). Parameters were assigned weakly informative priors, ${\alpha }_{y,s,a} \sim \mathrm{Normal}(0, 1/0.01)$ and ${p}_{g} \sim \mathrm{Beta}(1, 1)$, and estimated from three parallel Markov Chain Monte Carlo (MCMC) chains run for 30,000 iterations, thinned to retain every 100^th^ iteration after discarding the first 10,000 iterations as “burn-in”, with Just Another Gibbs Sampler (JAGS: Plummer 2003) via R package rjags (Plummer 2016) in R (version 4.0.3; R Core Team 2020). The estimated abundances of age-class 0 + , 1 + and sums of age 2 + to 5 + grayling, and age-class 0 + and larger trout, were used as life stage- and site-specific grayling and trout explanatory variables, respectively.

Except for number of days of low flow that decreased over time, there were no clear trends among the abiotic explanatory variables (Fig. 2). In contrast, mean macroinvertebrate biomass and mean adult grayling abundance decreased over time, and mean juvenile trout abundance increased over time, among the biotic explanatory variables (Fig. 2).

### Growth model

We extended a von Bertalanffy growth model to test the influence of life stage-specific effects of abiotic and biotic explanatory variables (Fig. 2) on expected length at age (i.e., somatic growth) of age 0 + to 5 + grayling caught at six sites across the 17-year sampling period. We defined the age 0 + grayling growth period in year *y* from their emergence in spring through to capture during autumn sampling (i.e., April to September/October in year *y*). For ages 1 + to 5 + , the growth period was defined as the 12-month period since the previous autumn sampling (i.e., September/October in year *y* – 1 to September/October in year *y*). These definitions allowed us to work with observed lengths rather than back-calculating length from scales, which can introduce additional uncertainties in the length-at-age estimation process (Gregory et al. 2018).

We allowed the explanatory variables to affect growth increments (representing their influences on energy acquisition: Matthias et al. 2018) of grayling life stages differently, where life stage, $l$, was defined as juvenile (age 0 + ; $l$ = 1), sub-adult (age 1 + ; $l$ = 2) or adult (ages 2 + to 5 + ; $l$ = 3). These life stages were designed – in part – to represent major ontogenetic shifts in sexual maturity (Lester et al. 2004). The expected juvenile (*a* = 1) length in year *y* and site *s* was estimated by

$$\mu_{y,s,a = 1} = L_{\infty } \times \left( {1 - e^{{ - K\left( {t_{a = 1} - t_{0} } \right)}} } \right) \times e^{{\theta_{l = 1} {X}_{y,s,l = 1} }} ,$$

where *y* = 1, …, 17 (representing years 2003–2019), *s* = 1, …, 6 (representing the six survey sites), ${L}_{\infty }$ is the asymptotic length of the oldest grayling in this population (i.e., the mean of age 5 + adults), $K$ is the Brody growth coefficient, ${t}_{a}$ is the age at current length, ${t}_{0}$ is the theoretical age when length is 0 mm, and ${\theta }_{l}={\beta }_{l,1},{\beta }_{l,2},...,{\beta }_{l,M}$ is a vector of coefficients relating the influences of $M$ stage-, year- and site- specific explanatory variables ${X}_{y,s,l}={\chi }_{y,s,l,1},...,{\chi }_{y,s,l,M}$ on the expected year-, site- and age-specific length, ${\mu }_{y,s,a}$. The expected length of subsequent age-classes *a* = 2, …, 6 (representing age 1 + to 5 + fish) was estimated by

$$\mu_{y + 1,s,a} = \left( {L_{\infty } - \mu_{y,s,a - 1} } \right) \times \left( {1 - e^{{ - K\left( {t_{a} - t_{0} } \right)}} } \right) \times e^{{\theta_{l} {X}_{y + 1,s,l} }} ,$$

where ${X}_{y+1,s, l}$ are again stage-, year- and site- specific explanatory variables, and $l$ = 2 for age-class 2 and $l$ = 3 for age-classes 3–6. This model structure resulted in coefficient estimates for juvenile and sub-adult life stages, each represented by a single age-class (1 and 2, respectively), and coefficient estimates for the adult life stage that represented multiple age-classes 3–6. To allow us to estimate expected length of age-classes *a* = 2, …, 6 in 2004, we specified prior distributions for ${\mu }_{y,1:6,a}$ in 2003 (Table 2). Model parameters were estimated from individual lengths by minimising the likelihood:

$$L_{i} { }\sim {\text{ Normal}}\left( {\mu_{y,s,a} ,} \right)$$

where *L* is the length of individual *i* that was captured in year *y*, in site *s* and at age-class *a*, and $\epsilon$ is an independent and identically distributed error term.

**Table 2:** Prior distributions assigned to parameters estimated in the grayling growth model

Parameter	Prior distribution
${L}_{\infty }$	$\sim \mathrm{Normal }(0, 0.001)$
$K$	$\sim \mathrm{Gamma }(1, 1)$
${t}_{0}$	$\sim \mathrm{Normal }(0, 0.001)$
$\epsilon$	$\sim \mathrm{Gamma }(0.01, 0.01)$
${\beta }_{l,M}$	$\sim \mathrm{Normal }(0, 0.001)$
${\mu }_{y,1:6,a}$	$\sim \mathrm{Normal }(0, 0.001)$

### Model evaluation and fitting

We evaluated the model performance by simulating grayling length data, structured to represent these study data (i.e. 17 years, six sites, six age-classes), generating model parameters and coefficient effects, and compared the model parameter estimates with those used to simulate the data. The full annotated code used to generate data and run the model simulation is provided in the supplementary material. We repeated this exercise with multiple randomly simulated datasets. Overall, the model returned reasonable estimates of grayling length, parameter values, and MCMC chain convergence (Figs. S4a, b). We did simulations because we had developed a bespoke and complex statistical model, and we wanted to be confident that the model could return true model parameters from multiple simulated datasets.

Prior to model fitting for inference, we used pairwise Pearson’s correlations to ensure life stage-specific explanatory variables were sufficiently statistically independent and non-collinear. We identified high positive correlation (*r* ≥|0.7|; Dormann et al. 2012, Fig. S5) between the variables ‘mean temperature (spring–autumn)’ and ‘number of days of high temperature (spring–autumn)’. Of the two variables, we chose to retain ‘mean temperature’ as this was considered a more fundamental influence of grayling growth (e.g., Mallet et al. 1999), and captured the recent trend of higher-than-average temperatures (Fig. 2a, b). To compare the effects of explanatory variables measured on different scales, all explanatory variables were *z*-standardised prior to analyses by subtracting their mean and dividing by their standard deviation.

We assigned weakly informative priors to all model parameters (Table 2) and fitted the growth model with JAGS via rjags in R, with inferences again drawn from three parallel MCMC chains run for 30,000 iterations, thinned to retain every 100^th^ iteration after discarding the first 20,000 iterations as ‘burn-in’.

### Model simplification

To test the hypothesised influences of our explanatory variables on each life stage, we simplified the growth model iteratively. Firstly, we fitted a saturated ‘juvenile model’ that included all juvenile-specific explanatory variables, while omitting those for sub-adults and adults, and simplified the model iteratively by each time removing explanatory variables whose estimated effect intercepted zero. We then extended the model retaining the important juvenile-specific explanatory variables to include a saturated ‘sub-adult model’, which included all sub-adult-specific explanatory variables, while still omitting those for adults, and simplified the model as before. We repeated this for a saturated ‘adult model’, which included the important explanatory variables specific to juveniles and sub-adults (Table S1).

The final simplified ‘adult model’ was considered the best fitting ‘full model’, but as uncertainty in the estimated effects of stage-specific explanatory variables can propagate throughout the model and thereby influence effects of other variables (Letcher et al. 2015), we further explored the ‘full model’. To verify that the model simplification procedure had retained the explanatory variables most important for describing expected length at each life stage, we compared the ‘full model’ to three ‘candidate full models’ that were the ‘full model’ with the re-addition of the last explanatory variable to be omitted for each life stage (Table S2). We compared these models using leave-one-out cross validation (LOO) implemented in the R package loo (Vehtari et al. 2019) selecting the most parsimonious model with the lowest LOO Information Criterion (LOOIC) as the model for inference (Table S3).

Finally, we considered the influence of recaptured individuals in our analysis. Of 5,602 observed lengths, 853 were from recaptured individuals. While we acknowledge that individual traits, such as metabolic rate, can influence growth potential (Rosenfeld et al. 2015), few individuals were recaptured and measured more than once (< 2.5% of the total sample size). Given the structure of our model, i.e. stratified across age, site and years, and the consequently relatively small number of recaptured vs non-recaptured individuals per stratification, any possible effects of non-independence between measures on recaptures were considered likely to be negligible. Nevertheless, this was tested by fitting the best performing ‘full model’ to data omitting lengths measured for all recaptures. As all but one of the covariate effects did not change in strength or direction (Table S2), we proceeded to use the ‘full model’ fit to all data for inference. For the final model, we verified convergence of the posterior distribution by calculating the Gelman-Rubin diagnostic statistic for each estimated parameter (values < 1.1 suggest convergence and well-mixing MCMC chains) and visually inspecting the trace plots to assess the mixing of the chains (Table S4, Fig. S6).

## Results

**Fig. 3:**
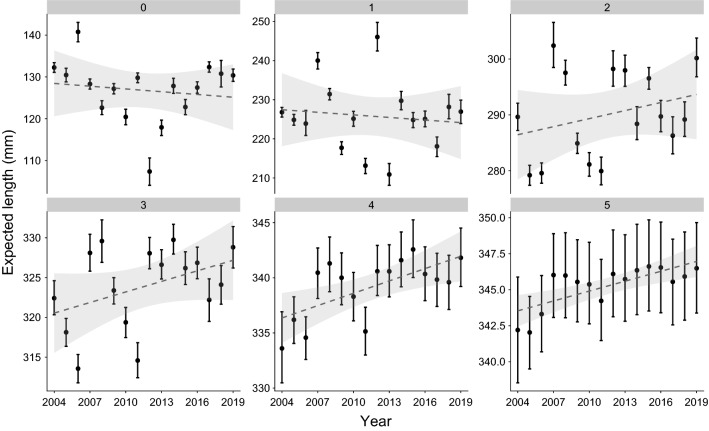
Mean expected grayling length at age (0 + to 5 +) as a function of the explanatory variables retained in the final model, averaged across site for each year. Points are the mean estimate and errorbars show the 95% Bayesian credible intervals. A linear Year term (mean effect and associated uncertainty shown in grey dashed line and shaded area) was added post-model fitting to visualise any temporal trend in expected length at age

**Fig. 4:**
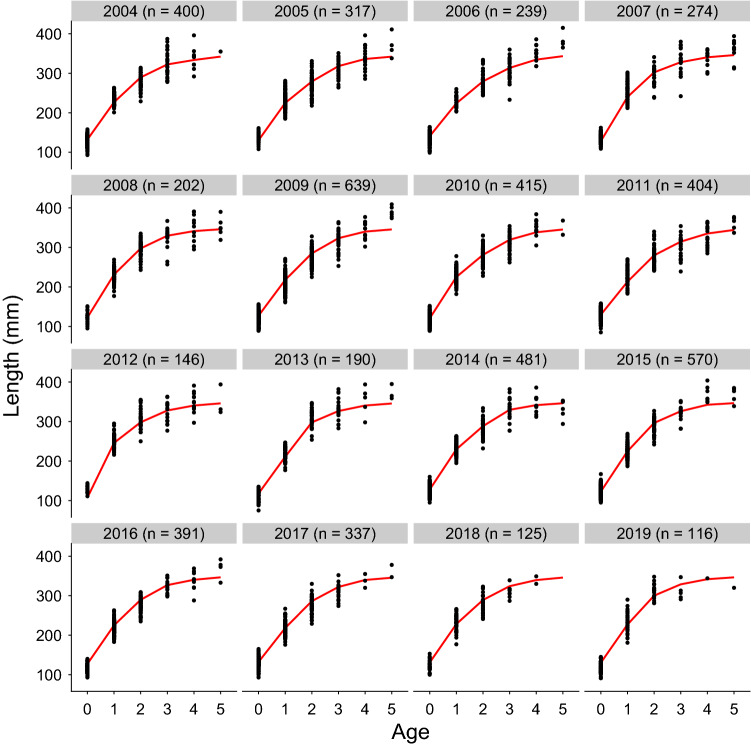
Model estimated length at age compared to empirical length at age. Mean length at age estimated as a function of the explanatory variables retained in the final model, averaged across site for each year is shown as a red line alongside empirical individual length data displayed as black points and the sample size shown in the plot label

Empirical support for each ‘candidate full model’ compared to the ‘full model’ was negligible and so the ‘full model’ was selected for inference (Table S3). The mean expected length of juvenile (0 +) and sub-adult (1 +) grayling was variable, but with a weak temporal decline (Fig. 3). In contrast, the mean expected length of adult grayling (ages 2 + to 5 +) increased over time (Fig. 3). Model estimated length at age was comparable to the empirical length at age for all years and ages, although expected mean length of age 5 + grayling appeared to be underestimated by the model in some years (Fig. 4). Grayling grew most rapidly in younger age-classes (between ages 0 + and 2 +) before slowing with the assumed onset of maturity (Fig. 4). Mean (and credible interval) estimates of the growth parameters were: ${L}_{\infty }$ = 347.61 (344.54, 351.39), $K$= 0.14 (0.12, 0.16) and ${t}_{0}$ =  – 2.27 ( – 2.74,  – 1.94).

**Fig. 5:**
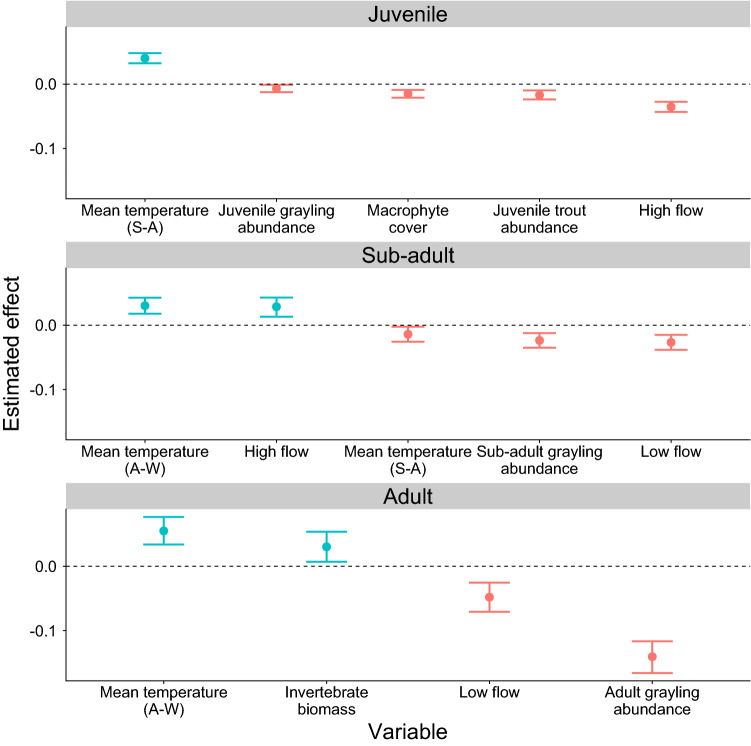
Coefficient estimates of the explanatory variables retained in the final model judged to influence expected length at age for each grayling life stage. Points are the mean estimated effect size, errorbars are the 95% Bayesian credible intervals, and the direction of the effect is coloured (blue is positive, red is negative). All variables relate to the main growing period during spring to autumn (S–A) with the exception of mean temperature during autumn–winter (A–W)

**Fig. 6:**
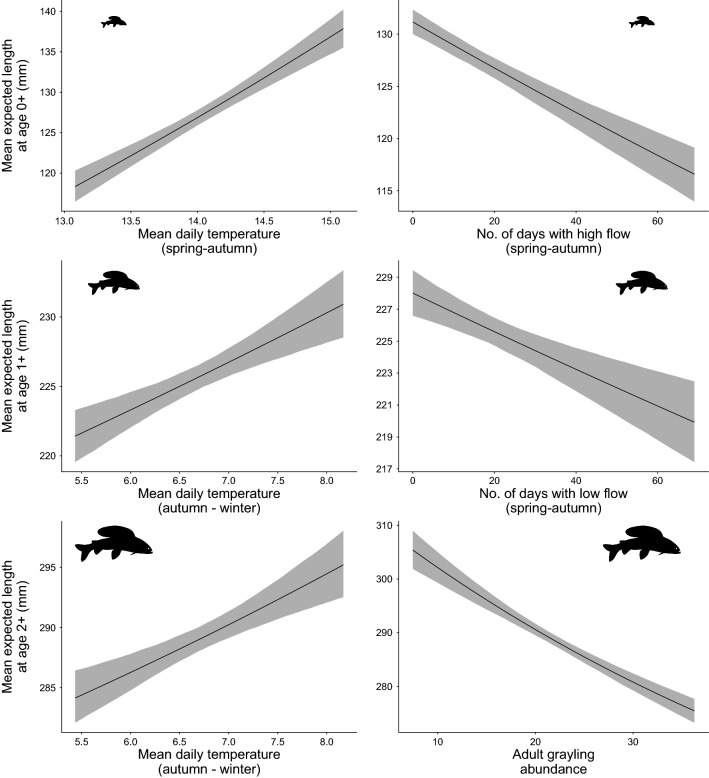
Marginal effects of the strongest positive (left-hand panels) and negative (right-hand panels) influences on expected grayling length at age 0 + (juvenile; top row), age 1 + (sub-adult; middle row) and age 2 + (representing adult; bottom row). The line and shaded area represents the mean (and 95% credible interval) effect of the explanatory variable whilst holding the effects of all other retained variables and the mean expected length of age of the previous age-class constant

Overall, the strength of the effects of abiotic and biotic explanatory variables on grayling growth was strongest among the adult age classes; their effects on juvenile and sub-adult grayling were comparatively weaker and often similar (Fig. 5). Grayling abundance had a consistent negative influence on expected length at each life stage, with the strongest effect on adult grayling (Fig. 5). For example, mean expected length of age 2 + grayling declined from > 300 mm to around 280 mm when adult abundance tripled from 10 to 30 individuals (Fig. 6). Temperature had the most positive influence at every life stage, albeit in different growing periods (Figs. 5, 6). Mean temperature during spring–autumn had a positive influence on juvenile expected length, but a negative influence on sub-adult expected length, contrary to our hypotheses (Fig. 5). In contrast, mean temperature during autumn–winter had a positive influence on sub-adult and adult expected length (Fig. 5).

Both sub-adult and adult expected length were negatively influenced by low flow (Fig. 5) with sub-adult expected length declining from 228 to 221 mm as days of low flow increased from 0 to 60 days (Fig. 6). Furthermore, juvenile expected length was negatively influenced by macrophyte cover, juvenile trout abundance and, particularly, high flow, for which an increase from 0 to 60 days of high flow resulted in a decrease of 10 mm in expected length (Figs. 5, 6). Sub-adult and adult expected length were positively influenced by high flow and invertebrate biomass, respectively (Fig. 5).

Contrary to our hypotheses, there were no discernible effects of large trout abundance on sub-adult and adult expected length, invertebrate biomass on juvenile and sub-adult expected length, low flow on juvenile expected length, macrophyte cover on sub-adult expected length and mean temperature during spring–autumn on adult expected length, therefore, these variables were omitted during the model simplification.

## Discussion

A wide range of abiotic and biotic variables can influence the age- and stage-specific growth of riverine fishes, and understanding these relationships is essential for identifying adequate management actions to ameliorate depleted populations. The results presented here revealed that interannual variations in grayling length at age were described by annual- and site-specific abiotic and biotic explanatory variables, and – importantly – that their influence differed by age and life stage. Notably, we confirm a number of a priori hypothesised life stage specific growth responses to increased temperature and extreme discharge events during the main growth period, which is especially pertinent for safeguarding habitats for all grayling life stages under future climate change scenarios. Furthermore, our results emphasise the merits of expanding explanatory variables tested to include biotic influences, such as habitat cover and competitor and prey abundances, all of which were influential in describing grayling growth in distinct life stages. Together, our findings highlight how different life stages can respond to the same conditions differently, thereby underscoring the importance of considering life stage specific requirements, perhaps due to ontogenetic shifts in sexual maturity (Lester et al. 2004), for the effective management of grayling populations in the face of changing environmental conditions.

We found that stage-specific grayling abundance negatively influenced expected length at each life stage, but only juvenile expected length was influenced by trout abundance. These findings confirmed our hypotheses suggesting that intraspecific competition exerts a large influence on grayling growth, although, contrary to our hypotheses, interspecific competition has little effect on older grayling growth. Specifically, juvenile grayling growth was lower when juvenile trout abundance was high, presumably because they compete for limiting resources. A recent experiment investigating competitive interactions between juvenile hatchery salmonids found that grayling fed less when in sympatry with brown trout or Atlantic salmon and displayed the most aggressive behaviour of all the species towards competitors (Hagelin and Bergman 2021). In our study, juvenile grayling in sites and years with higher competitor abundance might have had less growth potential through expending more energy defending territory and/ or competing for (and losing out on) food resources. However, juveniles were relatively weakly affected by the negative influence of conspecific abundance, which appeared to intensify through the succession of grayling life stages. This might represent ontogenetic shifts in behaviour, as juvenile grayling often form shoals that can be advantageous for foraging success and predator avoidance (Hart et al. 2014; Watz et al. 2020). These benefits might moderate the potential negative impacts of increased conspecific abundance on juvenile grayling growth, hence the weaker response relative to older life stages. Although evidence for competition between trout and grayling in natural conditions is limited, our findings support suggestions that interspecific competition decreases in intensity as individuals grow and distinct niches or interactive segregation develops (Greenberg et al. 1996; Ibbotson et al. 2001).

Spring to autumn temperature had contrasting influences on juvenile and sub-adult grayling expected length, which were positive for juveniles and negative for sub-adults. Temperature had the strongest positive influence on juvenile expected length, with a 1.5 °C rise in mean air temperature from 13.5 °C to 15 °C associated with an increase in expected length at age 0 + from 124 to 140 mm. This is consistent with our hypothesis and previous findings that summer temperature is a fundamental influence on juvenile growth of both European grayling (Mallet et al. 1999) and Arctic grayling (*Thymallus arcticus*) (Deegan et al. 1999). The opposite finding for sub-adult growth contradicts our hypothesis and suggests that spring to autumn temperatures increased beyond those optimal for older grayling. Physiological processes, such as respiration, metabolism, and reproduction are more costly in larger sized individuals, particularly in warming temperatures (Ohlberger, 2013; Rosenfeld et al. 2015), thus we might have also expected adult growth to be strongly, negatively influenced by spring to autumn temperature. It is possible that adults were more successful in utilising or competing for thermal refuges, such as deeper pools. Interestingly, temperature outside of the main growing period had the strongest positive influence on sub-adult and adult expected length, confirming our hypotheses and suggesting that growth opportunities during autumn to winter were not trivial; an increase in mean air temperature from 5.5 °C to 8 °C related to an increase in mean expected length of around 10 mm in ages 1 + and 2 + grayling. Warmer winters could therefore provide a valuable growth period for older grayling, perhaps in preparation for spring spawning, particularly if they follow relatively warm and poor summer growing conditions.

Our results confirmed the hypothesised negative influence of low flow on sub-adult and adult growth. This suggests that increases in the intensity and frequency of low discharge events forecast under climate-change will be an important stressor on European grayling growth, as well as their population dynamics, including recruitment (Bašić et al. 2018), and sub-adult and adult survival rates (Marsh et al. 2021). Low summer discharge can reduce the effective habitat areas (O’Brien and Showalter, 1993), and foraging opportunities by diminishing prey resource (Dewson et al. 2007) and prey encounter rates (Nislow et al. 2004), all of which increase their inter- and intra-specific competitive interactions and could contribute to reduced growth. The pronounced influence of warmer temperatures on older grayling growth might be due to increased metabolic demands of fish maintaining swimming positions in lower discharge (Deegan et al. 1999). Correspondingly and opposite to our hypothesis, high flow had a positive influence on sub-adult expected length, yet a hypothesised negative influence on juvenile expected length, a finding consistent with a study on Arctic grayling (Deegan et al. 1999). Whereas higher discharge is likely to increase the amount of invertebrate drift prey and encounter rates with prey (O’Brien and Showalter 1993), juvenile grayling might be less physically able to withstand increased discharge and capitalise on these resources, and growth potential might reduce if fish are forced out of optimum habitat into flow refuges (Deegan et al. 1999). The finding that both infrequent and frequent high flow events are negative for the growth of successive grayling life stages highlights the requirement for maintaining heterogeneous habitat with variable discharge rates and refuges that provide shelter from both extreme discharges and high water temperatures.

Summer macrophyte cover negatively influenced juvenile grayling expected lengths, as hypothesised, and could represent reduced growth potential as plant cover decreases their preferred benthic feeding habitat (Ibbotson et al. 2001). This finding corresponds with the negative influence of macrophyte cover on juvenile grayling survival (Marsh et al. 2021), suggesting that, while the precise mechanism(s) for these relationships remain elusive, instream vegetation cover could be reduced in management programmes to ameliorate depleted grayling populations. Macroinvertebrate biomass, representing the putative prey resource, was only influential on adult grayling expected length. As resource demands scale allometrically with body size (Brown and Maurer 1986), growth of mature adults might be more sensitive to inter-annual variation in prey biomass. Alternatively, this finding could be indicative of our study system, a relatively productive a chalk stream, that provides relatively abundant macroinvertebrate food resources year-round, which are rarely limiting (Berrie 1992). Thus, food resources during spring to autumn might be less limiting in the context of our study compared to species with shorter growing periods, such as Arctic grayling in tundra streams, of which growth is highly influenced by food availability (Deegan et al. 1997, 1999).

As with many field studies, we were limited by data availability and, as such, we made several assumptions. We assumed a constant age of maturation at age 2 + (Ibbotson et al. 2001) and an even sex-ratio in the population, based on a fecundity study referenced in Ibbotson et al. (2001) in which 36 grayling caught in the Wylye were dissected to confirm sex and maturity (Ibbotson, unpublished data). However, it is acknowledged that these factors can affect grayling growth. Specifically, upon maturation, male grayling are recorded to grow faster than females, possibly as egg production in salmonids is costly so females divert more resources from somatic to gonadal growth (Woolland and Jones 1975; Fleming 1998). Furthermore, maturation in species with indeterminate growth is often best described by body size, rather than distinct age-classes (Ohlberger 2013). Consequently, faster growth in young individuals can lead to maturation at a younger age (Tréhin et al. 2021). Understanding how changes to growth can influence population demographics through age of maturation and associated fitness could therefore be an interesting avenue of further study. Additionally, the explanatory variables tested here were not exhaustive and were limited to available data; other variables could also influence grayling growth. For example, changes in predator abundances and/or diet could partly explain the observed variation in mean length at age between age-classes and over time, but we could not test this because we did not follow individuals and we do not have data on predator abundance (beyond trout) or predator diet. Similarly, exploring growth seasonally, rather than by autumn–winter and spring–summer as here, would better account for any seasonal changes in growth, which could be fruitfully related to growth increments read from scale samples.

Despite these considerations, we argue that our approach successfully identified variables affecting stage-specific grayling growth from among a suite of hypothesised influential abiotic and biotic variables. Our findings suggest that mean expected length of juvenile and sub-adult grayling has declined slightly during the study period, which could result in reduced survival or fecundity at later life stages (Barneche et al. 2018; Korman et al. 2021). Indeed, survival probabilities for adult age-classes in this population have declined during the same time-series (Marsh et al. 2021). Lower adult abundances are therefore likely to be a strong driver of the temporal increase in mean expected length of adults found in this study. This positive response to low conspecific abundances could help to sustain the grayling population by producing larger adults with improved survival and reproductive potential, thus ensuring their persistence. It is an important next step to quantify the influence of reduced growth in younger grayling on survival in subsequent ages. This would also allow us to determine to what extent abiotic and biotic factors are acting directly on survival or indirectly through growth-mediated survival, further developing our insight into drivers of grayling population dynamics.

Relative to other ecosystems, freshwater habitats continue to be disproportionally threatened by anthropogenetic impacts (Reid et al. 2018), and climate change is driving warmer water temperatures and more extreme discharge events (Huml et al. 2020; Gudmundsson et al. 2021). Such environmental changes have been associated with decreases in the average sizes of many organisms, from crop plants to fishes (Sheridan and Bickford 2011), including salmonids (e.g., Gregory et al. 2017). Moreover, since body size is a key determinant of survival, especially among fish (Sogard 1997), including salmonids (Gregory et al. 2019), decreases in body size could lead to declines in the size of at-risk populations. It is therefore imperative to understand better the life-stage specific vulnerabilities of grayling to changing environmental conditions. This will feed into fishery management strategies aimed at protecting and conserving grayling stocks under the predicted future climatic and hydrological conditions. For instance, when finding that environmental changes affect the growth of life stages differently, management efforts should aim to maintain sufficient habitat diversity not only to support ontogenetic shifts in habitat use, but also to allow for potential adaptations in habitat use under future, potentially inclement, change. Similarly, being able to identify how a specific environmental stressor affects growth across multiple life stages presents the opportunity to enhance growing conditions for the entire population with minimum intervention.

## Supplementary Information

Below is the link to the electronic supplementary material.

Supplementary file1 (DOCX 1904 KB)

## Data Availability

The data are available from the authors upon reasonable request.

## Code availability

The annotated code used to simulate data and fit the growth model is provided in the Supplementary Material.
