# Supplementary material for: Density-dependence and environmental variability have stage-specific influences on European grayling growth
Source: Oecologia. 2022 May 4;199(1):103–17. doi: 10.1007/s00442-022-05163-2 (PMC9119903; doi:10.1007/s00442-022-05163-2)
Supplement: Supplementary file 1 — Supplementary file1 (DOCX 1904 KB) [file 442_2022_5163_MOESM1_ESM.docx]

**Supporting information**

Density-dependence and environmental variability have stage-specific influences on European grayling growth

**Jessica E. Marsh^1, 2, 3*^, Richard J. Cove^4^, J. Robert Britton^2^, Robert G. Wellard^5^, Tea Bašić^6^, Stephen D. Gregory^1,3^**

*^1^Salmon and Trout Research Centre, Game & Wildlife Conservation Trust, The River Laboratory, Wareham, Dorset, UK*

*^2^Department of Life and Environmental Sciences, Faculty of Science and Technology, Bournemouth University, Poole, Dorset, UK*

*^3^Centre for Environment, Fisheries and Aquaculture Science (Cefas), Weymouth, Dorset, UK^4^Natural Resources Wales/Cyfoeth Naturiol Cymru, Buckley, Flintshire, UK*

*^5^The Piscatorial Society, Wiltshire, UK*

*^6^Salmon and Freshwater Team, Centre for Environment, Fisheries and Aquaculture Science (Cefas), Lowestoft, Suffolk, UK*

* Corresponding author


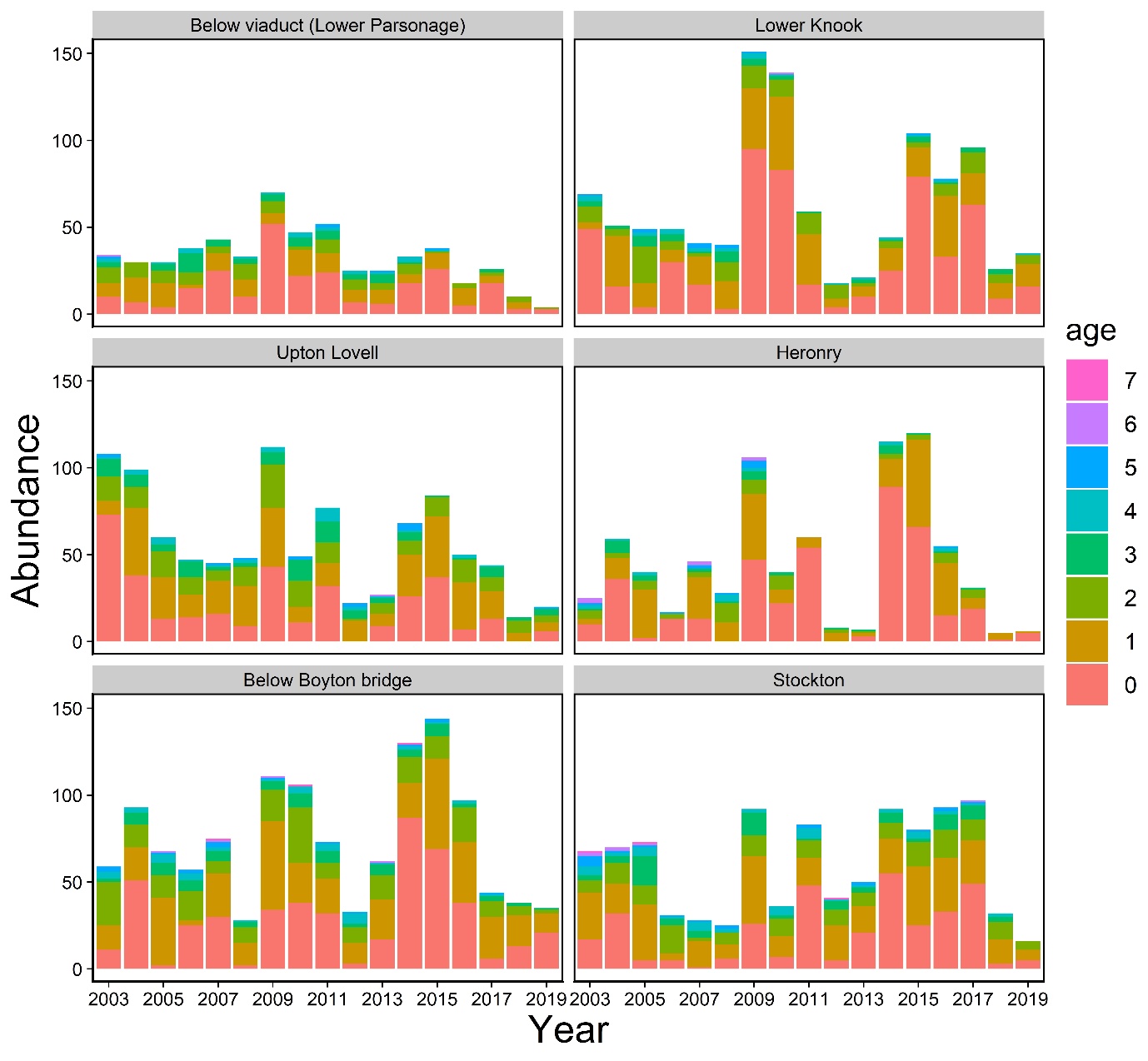


**Fig. S1.** Raw abundance counts of all ages of grayling caught in each site for each year of the study period. Sites are ordered from the most upstream site (Below viaduct – Lower Parsonage) to the most downstream site (Stockton).


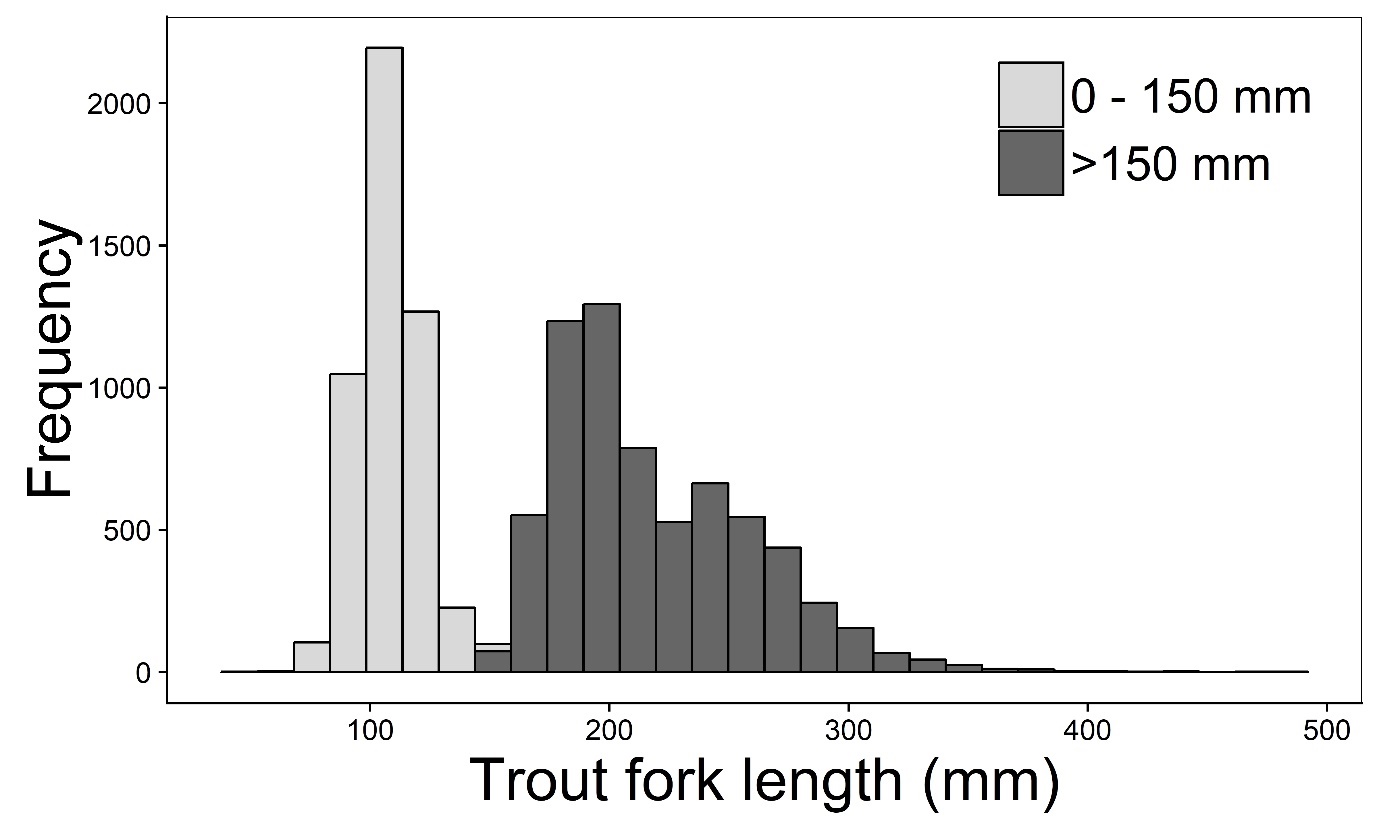


**Fig. S2.** Length-frequency histogram of trout caught across all sites and years of the study. Data are coloured to illustrate grouping of juvenile trout (0 – 150 mm) and older, large trout (> 150 mm).


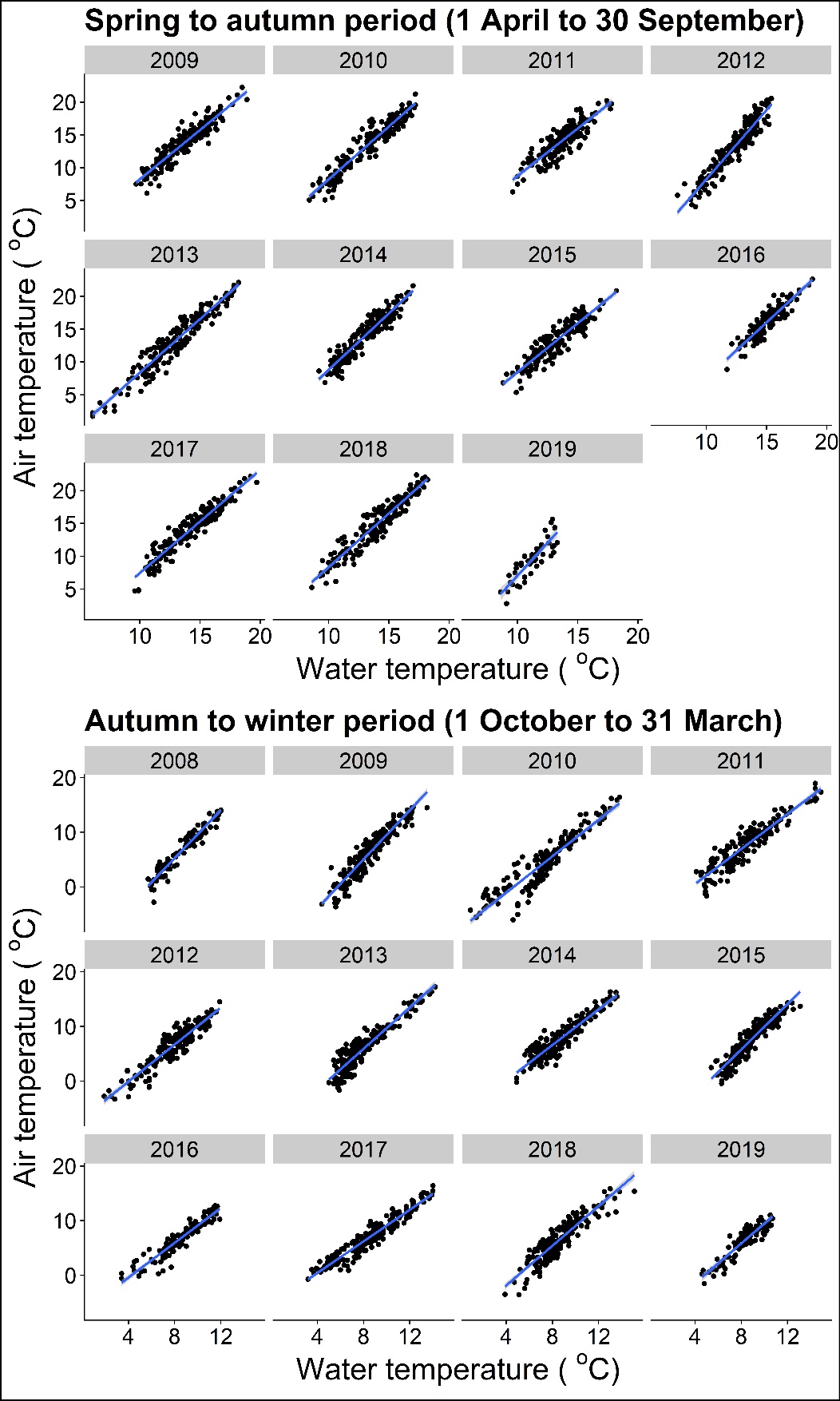


**Fig. S3**. Relationship between daily mean water temperature measured at South Newton and daily mean air temperature obtained from the E-OBS dataset for the spring to autumn and the autumn to winter periods (*r^2^* ranges between 0.87 – 0.90). Black points are the raw data and the blue line is the fitted linear regression.


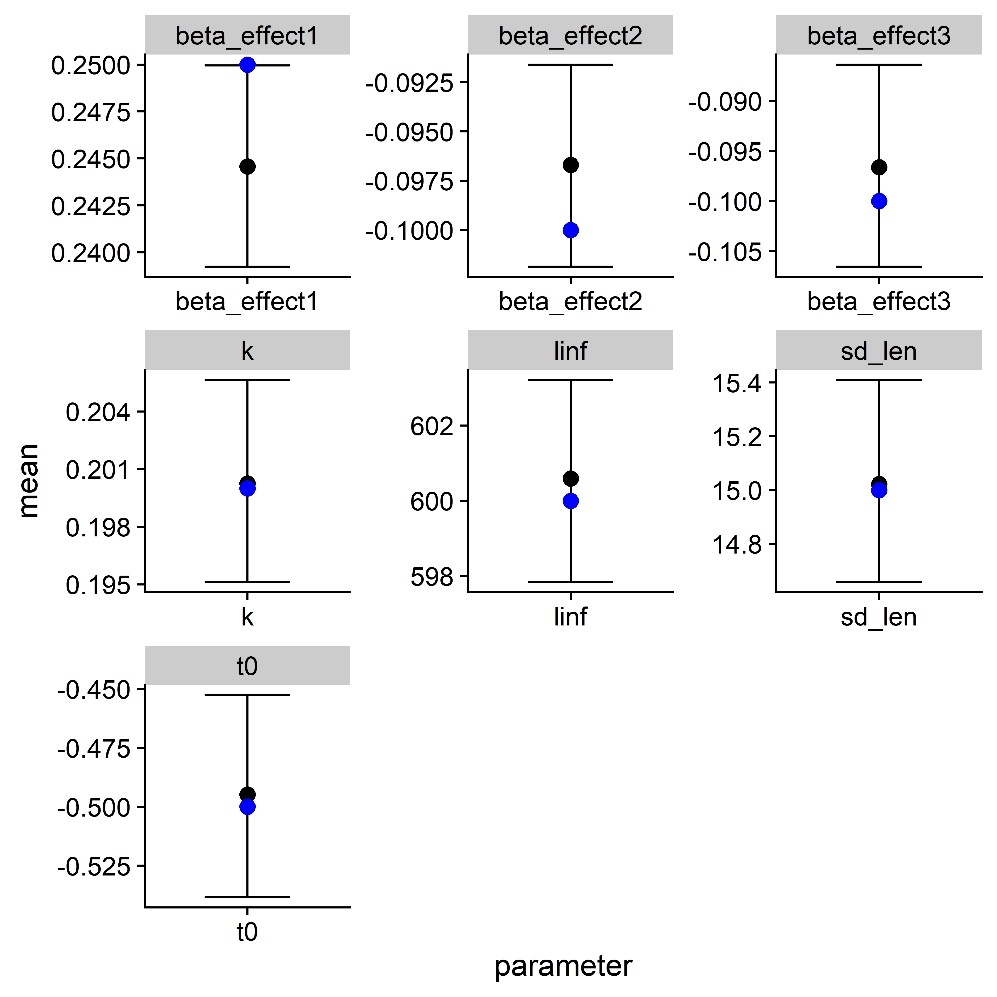

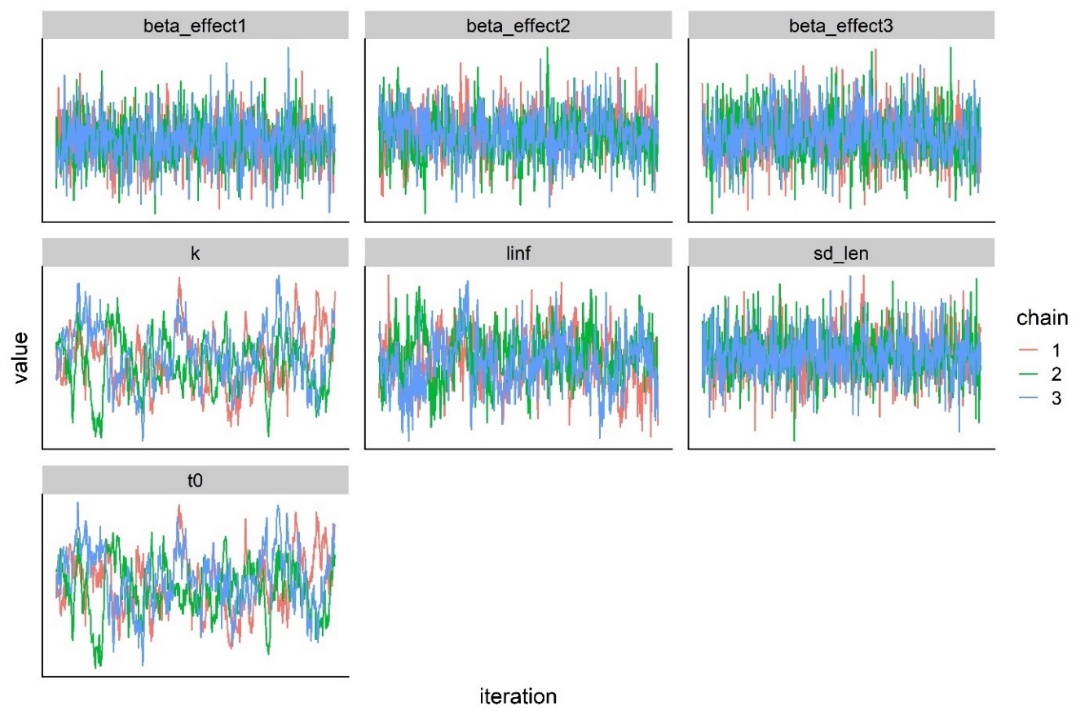


**Fig. S4a**. Example of a model simulation result: The top panel shows the mean and 95% credible intervals of the model estimated parameters (black) compared to the simulated parameters (blue). The code used to run the simulation, including generating the simulated parameters, is provided at the end of this document. The beta_effect parameters show the generated coefficient estimates; k, linf and t0 represent the generated growth parameters, and sd_len is the standard deviation of observed length. The bottom panel shows trace plots of the MCMC chains of model parameters. All Rhat values for estimated parameters < 1.1 indicating well-mixed chains.


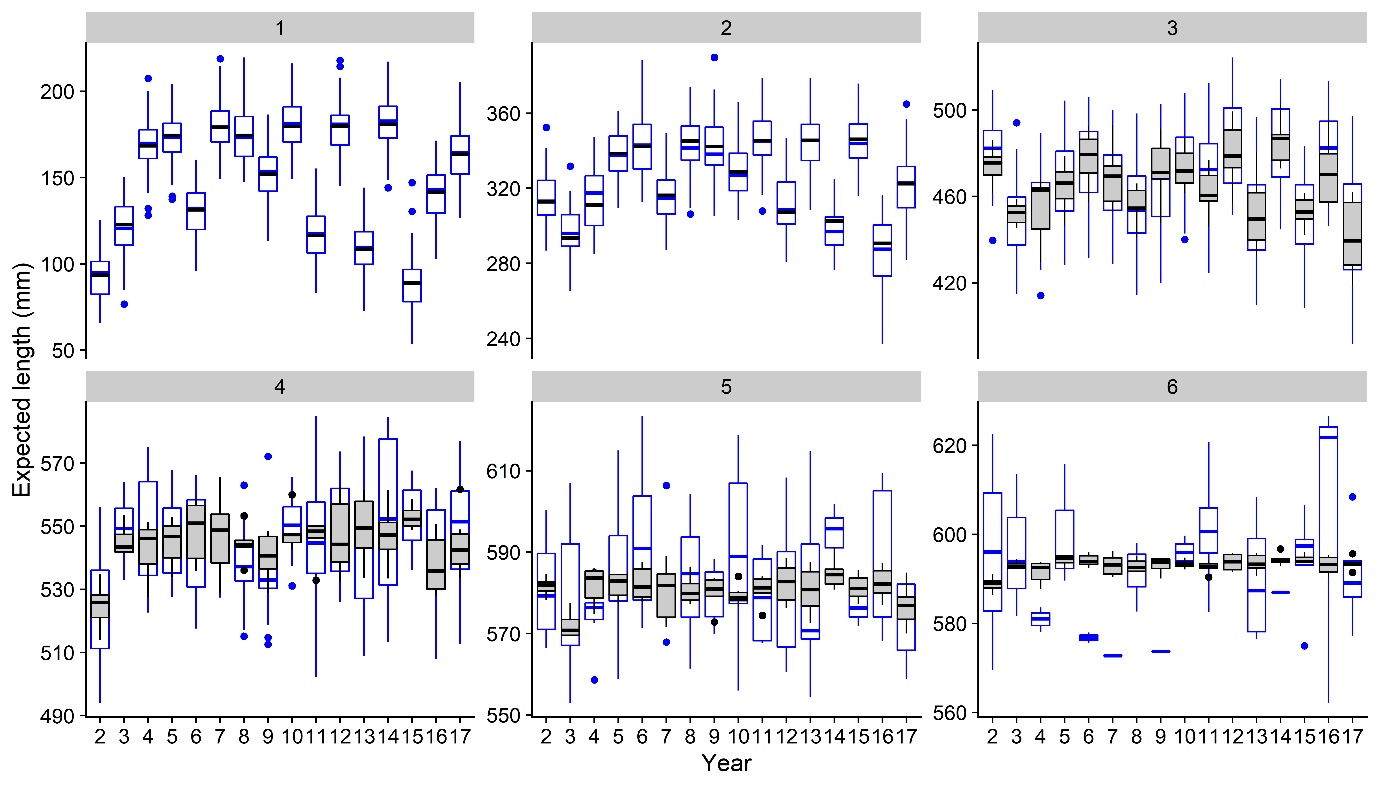


**Fig. S4b**. Example of a model simulation result: Summary of the expected length at age (1 to 6 representing ages 0+ to 5+) of the model estimates (black and grey) compared with the simulated data (blue and white). The code used to run the simulation, including generating the simulated expected length at age, is provided at the end of this document. Boxplots represent the median and interquartile range of expected length at age for each year.


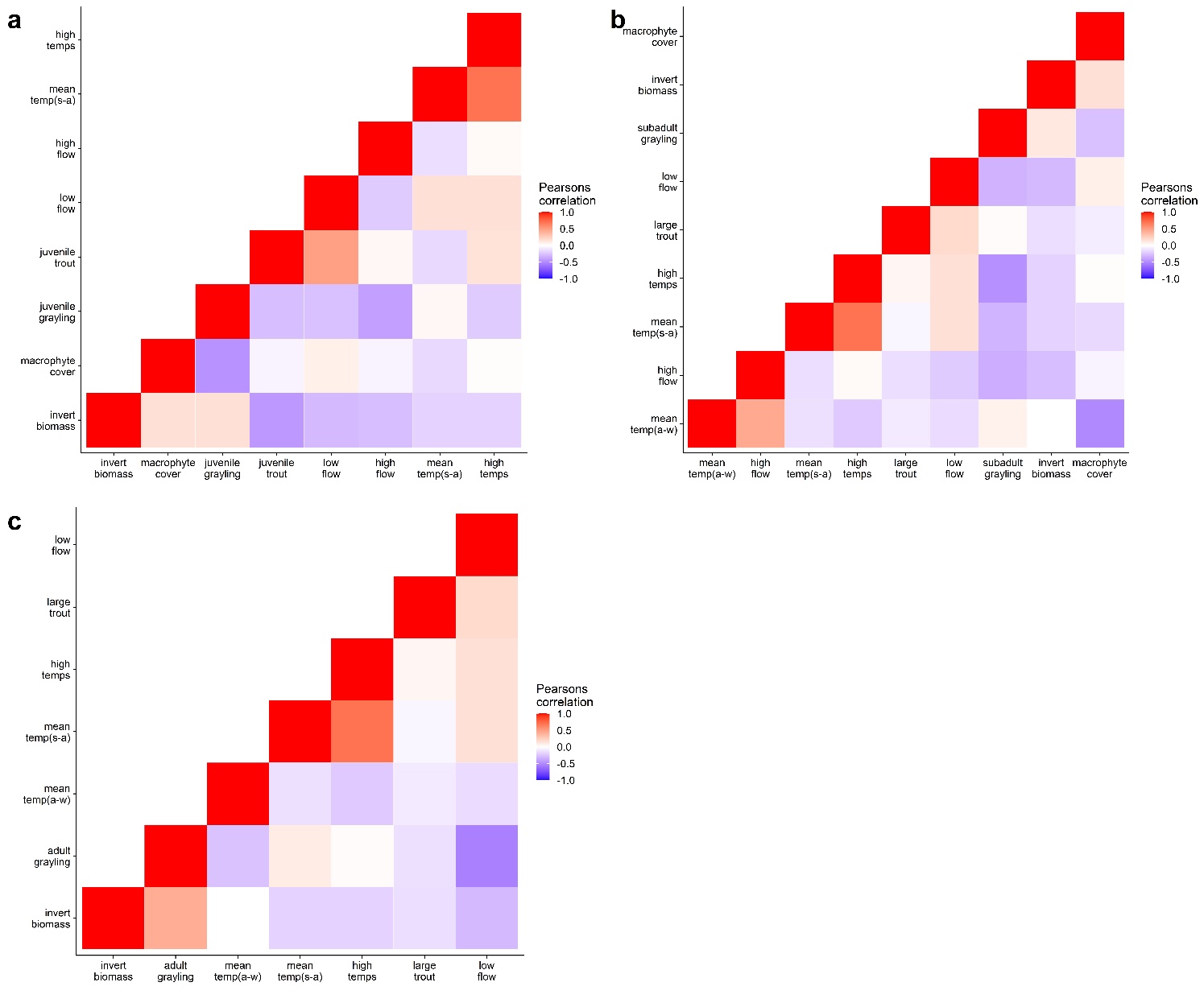


**Fig. S5**. Correlation matrix of explanatory variables tested in the growth model at a) juvenile, b) subadult and c) adult life-stages. All Pearson’s pair-wise correlations were < 0.7, except the relationship between mean temperature (spring-autumn) and number of high temperature events (*­r* ­= |0.7|).

**Table S1.** Model simplification process for each life-stage (the variable removed in each subsequent model fitting is highlighted in green): Mean = estimated mean effect size; C. I = Bayesian credible interval; n_eff = effective sample size; Rhat = potential scale reduction statistic or Gelman-Rubin statistic (values <1.1 suggest convergence), S-A and A-W in parenthesis represents the spring-autumn and autumn-winter temperature variable.

Juveniles

| **Model** | **Life-stage** | **Variable** | **Mean** | **2.5% C. I** | **97.5% C. I** | **n_eff** | **Rhat** |
| --- | --- | --- | --- | --- | --- | --- | --- |
| 1 – all variables | Juvenile | Juv grayling abundance | -0.01 | -0.02 | -0.01 | 509 | 1.00 |
|  |  | High flow | -0.04 | -0.05 | -0.04 | 558 | 1.01 |
|  |  | Invertebrate biomass | -0.01 | -0.01 | 0.00 | 600 | 1.00 |
|  |  | Low flow | -0.01 | -0.01 | 0.00 | 547 | 1.00 |
|  |  | Macrophyte | -0.01 | -0.02 | -0.01 | 641 | 1.00 |
|  |  | Mean temperature (S-A) | 0.05 | 0.04 | 0.05 | 849 | 1.01 |
|  |  | Juv trout abundance | -0.01 | -0.02 | 0.00 | 829 | 1.00 |
| 2 – all variables minus Low flow | Juvenile | Juv grayling abundance | -0.01 | -0.02 | -0.01 | 600 | 1.00 |
|  |  | High flow | -0.04 | -0.05 | -0.03 | 552 | 1.00 |
|  |  | Invertebrate biomass | 0.00 | -0.01 | 0.00 | 600 | 1.00 |
|  |  | Macrophyte | -0.01 | -0.02 | -0.01 | 635 | 1.00 |
|  |  | Mean temperature (S-A) | 0.05 | 0.04 | 0.05 | 600 | 1.00 |
|  |  | Juv trout abundance | -0.01 | -0.02 | 0.00 | 541 | 1.00 |
| 3 – final ‘juvenile’ model: all variables minus low flow and invertebrate biomass | Juvenile | Juv grayling abundance | -0.01 | -0.02 | -0.01 | 562 | 1.01 |
|  |  | High flow | -0.04 | -0.05 | -0.03 | 600 | 1.00 |
|  |  | Macrophyte | -0.02 | -0.02 | -0.01 | 600 | 1.01 |
|  |  | Mean temperature (S-A) | 0.05 | 0.04 | 0.05 | 600 | 1.00 |
|  |  | Juv trout abundance | -0.01 | -0.02 | 0.00 | 643 | 1.00 |

Subadults

| **Model** | **Life-stage** | **Variable** | **Mean** | **2.5% C. I** | **97.5% C. I** | **n_eff** | **Rhat** |
| --- | --- | --- | --- | --- | --- | --- | --- |
| 1 – all subadult variables (plus retained juvenile variables in grey) | Juvenile | Juv grayling abundance | -0.01 | -0.01 | 0.00 | 559 | 1.01 |
|  |  | High flow | -0.03 | -0.04 | -0.03 | 677 | 1.02 |
|  |  | Macrophyte | -0.02 | -0.02 | -0.01 | 671 | 1.02 |
|  |  | Mean temperature (S-A) | 0.04 | 0.03 | 0.05 | 600 | 1.00 |
|  |  | Juv trout abundance | -0.02 | -0.02 | -0.01 | 731 | 1.01 |
|  | Subadult | Subad grayling abundance | -0.04 | -0.05 | -0.03 | 543 | 1.04 |
|  |  | High flow | 0.02 | 0.01 | 0.04 | 600 | 1.03 |
|  |  | Invertebrate biomass | -0.01 | -0.02 | 0.01 | 687 | 1.00 |
|  |  | Low flow | -0.03 | -0.04 | -0.01 | 600 | 1.00 |
|  |  | Macrophyte | 0.01 | -0.01 | 0.02 | 600 | 1.00 |
|  |  | Mean temperature (S-A) | -0.02 | -0.03 | 0.00 | 600 | 1.00 |
|  |  | Large trout abundance | 0.01 | 0.00 | 0.02 | 795 | 1.00 |
|  |  | Mean winter (A-W) | 0.03 | 0.02 | 0.05 | 559 | 1.00 |
| 2 – all subadult variables minus macrophyte (plus retained juvenile variables in grey) | Juvenile | Juv grayling abundance | -0.01 | -0.01 | 0.00 | 501 | 1.00 |
|  |  | High flow | -0.03 | -0.04 | -0.03 | 794 | 1.00 |
|  |  | Macrophyte | -0.02 | -0.02 | -0.01 | 541 | 1.01 |
|  |  | Mean temperature (S-A) | 0.04 | 0.03 | 0.05 | 696 | 1.00 |
|  |  | Juv trout abundance | -0.02 | -0.02 | -0.01 | 600 | 1.01 |
|  | Subadult | Subad grayling abundance | -0.04 | -0.05 | -0.03 | 600 | 1.00 |
|  |  | High flow | 0.02 | 0.00 | 0.04 | 670 | 1.01 |
|  |  | Invertebrate biomass | 0.00 | -0.02 | 0.01 | 530 | 1.00 |
|  |  | Low flow | -0.03 | -0.04 | -0.01 | 518 | 1.00 |
|  |  | Mean temperature (S-A) | -0.02 | -0.03 | 0.00 | 494 | 1.00 |
|  |  | Large trout abundance | 0.01 | 0.01 | 0.02 | 600 | 1.00 |
|  |  | Mean temperature (A-W) | 0.03 | 0.02 | 0.04 | 600 | 1.00 |
| **Model** |  | **Variable** | **Mean** | **2.5% C. I** | **97.5% C. I** | **n_eff** | **Rhat** |
| 3 – all subadult variables minus macrophyte and trout abundance (plus retained juvenile variables in grey) | Juvenile | Juv grayling abundance | -0.01 | -0.02 | 0.00 | 404 | 1.00 |
|  |  | High flow | -0.03 | -0.04 | -0.03 | 600 | 1.00 |
|  |  | Macrophyte | -0.02 | -0.02 | -0.01 | 716 | 1.00 |
|  |  | Mean temperature (S-A) | 0.04 | 0.03 | 0.05 | 549 | 1.00 |
|  |  | Juv trout abundance | -0.02 | -0.02 | -0.01 | 1101 | 1.00 |
|  | Subadult | Subad grayling abundance | -0.04 | -0.05 | -0.03 | 368 | 1.00 |
|  |  | High flow | 0.02 | 0.01 | 0.04 | 548 | 1.00 |
|  |  | Invertebrate biomass | 0.00 | -0.02 | 0.01 | 403 | 1.00 |
|  |  | Low flow | -0.03 | -0.04 | -0.01 | 581 | 1.00 |
|  |  | Mean temperature (S-A) | -0.02 | -0.03 | 0.00 | 477 | 1.00 |
|  |  | Mean temperature (A-W) | 0.03 | 0.02 | 0.05 | 600 | 1.00 |
| 4 – final ‘subadult’ model: all subadult variables minus macrophyte, trout abundance and invertebrate biomass (plus retained juvenile variables in grey) | Juvenile | Juv grayling abundance | -0.01 | -0.02 | 0.00 | 557 | 1.01 |
|  |  | High flow | -0.03 | -0.04 | -0.03 | 559 | 1.00 |
|  |  | Macrophyte | -0.01 | -0.02 | -0.01 | 695 | 1.00 |
|  |  | Mean temperature (S-A) | 0.04 | 0.03 | 0.05 | 590 | 1.00 |
|  |  | Juv trout abundance | -0.02 | -0.02 | -0.01 | 565 | 1.00 |
|  | Subadult | Subad grayling abundance | -0.04 | -0.05 | -0.03 | 498 | 1.00 |
|  |  | High flow | 0.02 | 0.01 | 0.04 | 600 | 1.00 |
|  |  | Low flow | -0.02 | -0.04 | -0.01 | 600 | 1.00 |
|  |  | Mean temperature (S-A) | -0.02 | -0.03 | 0.00 | 548 | 1.00 |
|  |  | Mean temperature (A-W) | 0.03 | 0.02 | 0.04 | 680 | 1.00 |

Adults

| **Model** | **Life-stage** | **Variable** | **Mean** | **2.5% C. I** | **97.5% C. I** | **n_eff** | **Rhat** |
| --- | --- | --- | --- | --- | --- | --- | --- |
| 1 – all adult variables (plus retained juvenile and subadult variables in grey) | Juvenile | Juv grayling abundance | -0.01 | -0.01 | 0.00 | 406 | 1.01 |
|  |  | High flow | -0.04 | -0.04 | -0.03 | 673 | 1.01 |
|  |  | Macrophyte | -0.02 | -0.02 | -0.01 | 671 | 1.01 |
|  |  | Mean temperature (S-A) | 0.04 | 0.03 | 0.05 | 922 | 1.00 |
|  |  | Juv trout abundance | -0.02 | -0.02 | -0.01 | 660 | 1.00 |
|  | Subadult | Sub grayling abundance | -0.02 | -0.04 | -0.01 | 284 | 1.00 |
|  |  | High flow | 0.03 | 0.01 | 0.05 | 633 | 1.00 |
|  |  | Low flow | -0.03 | -0.04 | -0.02 | 600 | 1.00 |
|  |  | Mean temperature (S-A) | -0.01 | -0.03 | 0.00 | 821 | 1.00 |
|  |  | Mean temperature (A-W) | 0.03 | 0.02 | 0.04 | 770 | 1.00 |
|  | Adult | Ad grayling abundance | -0.14 | -0.16 | -0.11 | 600 | 1.01 |
|  |  | Invertebrate biomass | 0.03 | 0.00 | 0.05 | 711 | 1.00 |
|  |  | Low flow | -0.04 | -0.07 | -0.02 | 668 | 1.00 |
|  |  | Mean temperature (S-A) | -0.02 | -0.04 | 0.00 | 950 | 1.00 |
|  |  | Large trout abundance | 0.00 | -0.03 | 0.02 | 765 | 1.00 |
|  |  | Mean temperature (A-W) | 0.05 | 0.03 | 0.07 | 600 | 1.00 |
| 2 –all adult variables minus trout abundance (plus retained juvenile and subadult variables in grey) | Juvenile | Juv grayling abundance | -0.01 | -0.01 | 0.00 | 413 | 1.01 |
|  |  | High flow | -0.04 | -0.04 | -0.03 | 600 | 1.00 |
|  |  | Macrophyte | -0.02 | -0.02 | -0.01 | 592 | 1.00 |
|  |  | Mean temperature (S-A) | 0.04 | 0.03 | 0.05 | 562 | 1.00 |
|  |  | Juv trout abundance | -0.02 | -0.02 | -0.01 | 600 | 1.00 |
|  | Subadult | Sub grayling abundance | -0.03 | -0.05 | -0.02 | 294 | 1.00 |
|  |  | High flow | 0.02 | 0.01 | 0.03 | 651 | 1.00 |
|  |  | Low flow | -0.03 | -0.04 | -0.02 | 600 | 1.00 |
|  |  | Mean temperature (S-A) | -0.02 | -0.03 | 0.00 | 600 | 1.00 |
|  |  | Mean temperature (A-W) | 0.03 | 0.02 | 0.04 | 713 | 1.00 |
|  | Adult | Ad grayling abundance | -0.14 | -0.16 | -0.11 | 600 | 1.00 |
|  |  | Invertebrate biomass | 0.02 | 0.00 | 0.05 | 493 | 1.01 |
|  |  | Low flow | -0.04 | -0.06 | -0.02 | 600 | 1.01 |
|  |  | Mean temperature (S-A) | -0.03 | -0.04 | 0.00 | 600 | 1.00 |
|  |  | Mean temperature (A-W) | 0.05 | 0.03 | 0.07 | 600 | 1.00 |
| 2 –Full model: all adult variables minus trout abundance and mean temperature (S-A) (plus retained juvenile and subadult variables in grey) | Juvenile | Juv grayling abundance | -0.01 | -0.01 | 0.00 | 553 | 1.01 |
|  |  | High flow | -0.04 | -0.04 | -0.03 | 600 | 1.00 |
|  |  | Macrophyte | -0.02 | -0.02 | -0.01 | 546 | 1.00 |
|  |  | Mean temperature (S-A) | 0.04 | 0.03 | 0.05 | 600 | 1.00 |
|  |  | Juv trout abundance | -0.02 | -0.02 | -0.01 | 600 | 1.01 |
|  | Subadult | Sub grayling abundance | -0.02 | -0.04 | -0.01 | 454 | 1.01 |
|  |  | High flow | 0.03 | 0.01 | 0.04 | 618 | 1.01 |
|  |  | Low flow | -0.03 | -0.04 | -0.01 | 600 | 1.00 |
|  |  | Mean temperature (S-A) | -0.01 | -0.03 | 0.00 | 617 | 1.00 |
|  |  | Mean temperature (A-W) | 0.03 | 0.02 | 0.04 | 600 | 1.00 |
|  | Adult | Ad grayling abundance | -0.14 | -0.16 | -0.12 | 557 | 1.00 |
|  |  | Invertebrate biomass | 0.03 | 0.01 | 0.05 | 547 | 1.01 |
|  |  | Low flow | -0.05 | -0.07 | -0.03 | 633 | 1.01 |
|  |  | Mean temperature (A-W) | 0.06 | 0.03 | 0.08 | 817 | 1.00 |

**Table S2.** Full model fitting and comparison. To verify the model simplification, we fit a ‘full candidate model’ set with the addition of the variables that were last removed in the model simplification for each life-stage (Models 2 to 4) and compared the models with LOO cross-validation (Table S3). We also fit the full model to length data that excluded any lengths observed in recaptured individuals (Model 5) to assess the influence of including recaptures (*n* = 853) in the dataset (*n* = 5,602). S-A and A-W in parenthesis represents the spring-autumn and autumn-winter temperature variable.

| **Model** | **Variable** | **Life-stage** | | **Mean (95% CI)** | **n_eff** | **Rhat** |
| --- | --- | --- | --- | --- | --- | --- |
| 1 – full model | Grayling abundance  High flow | | Juvenile  Subadult  Adult  Juvenile  Subadult | -0.01 (-0.01, 0.00)  -0.02 (-0.04, -0.01)  -0.14 (-0.16, -0.12)  -0.04 (-0.04, -0.03)  0.03 (0.01, 0.04) | 553  454  557  600  618 | 1.01  1.01  1.00  1.00  1.01 |
|  | Invertebrate biomass  Low flow  Macrophyte cover | | Adult  Subadult  Adult  Juvenile | 0.03 (0.01, 0.05)  -0.03 (-0.04, -0.01)  -0.05 (-0.07, -0.03)  -0.02 (-0.02, -0.01) | 547  600  633  546 | 1.00  1.00  1.01  1.00 |
|  | Mean temperature (S-A) | | Juvenile | 0.04 (0.03, 0.05) | 600 | 1.00 |
|  | Trout abundance  Mean temperature (A-W)  Growth parameter ($L_{\infty}$)  Growth parameter ($K$)  Growth parameter ($t_{0}$) | | Subadult  Juvenile  Subadult  Adult  -  -  - | -0.01 (-0.03, 0.00)  -0.02 (-0.02, -0.01)  0.03 (0.02, 0.04)  0.06 (0.03, 0.08)  348.34 (345.00, 351.54)  0.14 (0.12, 0.15)  -2.34 (-2.66, -2.04) | 617  600  600  817  130  59  58 | 1.00  1.00  1.00  1.00  1.01  1.02  1.02 |
| 2 – full model with addition of the last juvenile variable that was removed (shown in bold) | Grayling abundance  High flow | | Juvenile  Subadult  Adult  Juvenile  Subadult | -0.01 (-0.01, 0.00)  -0.02 (-0.03, -0.01)  -0.14 (-0.16, -0.12)  -0.03(-0.04, -0.03)  0.03 (0.01, 0.04) | 391  445  600  600  645 | 1.00  1.00  1.00  1.00  1.00 |
|  | **Invertebrate biomass**  Low flow  Macrophyte cover | | **Juvenile**  Adult  Subadult  Adult  Juvenile | **0.00 (0.00, 0.01)**  0.03 (0.01, 0.05)  -0.02(-0.04, -0.01)  -0.05 (-0.07, -0.03)  -0.02 (-0.02, -0.01) | 459  668  562  600  459 | 1.00  1.00  1.00  1.00  1.00 |
|  | Mean temperature (S-A) | | Juvenile | 0.04 (0.03, 0.05) | 600 | 1.00 |
|  | Trout abundance  Mean temperature (A-W)  Growth parameter ($L_{\infty}$)  Growth parameter ($K$)  Growth parameter ($t_{0}$) | | Subadult  Juvenile  Subadult  Adult  -  -  - | -0.02 (-0.03, 0.00)  -0.02 (-0.02, -0.01)  0.03 (0.02, 0.04)  0.05 (0.03, 0.08)  348.45 (344.95, 351.48)  0.13 (0.12, 0.15)  -2.38 (-2.68, -2.05) | 644  600  657  600  116  69  70 | 1.00  1.00  1.00  1.00  1.02  1.03  1.02 |
| 3 – full model with addition of the last subadult variable that was removed (shown in bold) | Grayling abundance  High flow | | Juvenile  Subadult  Adult  Juvenile  Subadult | -0.01 (-0.01, 0.00)  -0.02 (-0.04, -0.01)  -0.14 (-0.16, -0.12)  -0.03 (-0.04, -0.03)  0.03 (0.02, 0.05) | 415  507  724  572  600 | 1.03  1.02  1.00  1.01  1.01 |
|  | **Invertebrate biomass**  Low flow  Macrophyte cover | | **Subadult**  Adult  Subadult  Adult  Juvenile | **0.01 (0.00, 0.02)**  0.03 (0.01, 0.05)  -0.02 (-0.04, -0.01)  -0.05 (-0.07, -0.02)  -0.01 (-0.02, -0.01) | 665  649  600  544  833 | 1.00  1.00  1.00  1.00  1.00 |
|  | Mean temperature (S-A) | | Juvenile | 0.04 (0.03, 0.05) | 575 | 1.00 |
|  | Trout abundance  Mean temperature (A-W)  Growth parameter ($L_{\infty}$)  Growth parameter ($K$)  Growth parameter ($t_{0}$) | | Subadult  Juvenile  Subadult  Adult  -  -  - | -0.01 (-0.02, 0.00)  -0.02 (-0.02, -0.01)  0.03 (0.02, 0.04)  0.06 (0.04, 0.08)  347.84 (344.70, 350.82)  0.14 (0.12, 0.15)  -2.29 (-2.63, -1.99) | 540  401  600  600  104  58  56 | 1.00  1.00  1.00  1.00  1.07  1.13  1.15 |
| 4 – full model with addition of the last adult variable that was removed (shown in bold) | Grayling abundance  High flow | | Juvenile  Subadult  Adult  Juvenile  Subadult | -0.01 (-0.01, 0.00)  -0.02 (-0.04, -0.01)  -0.14 (-0.16, -0.11)  -0.04 (-0.04, -0.03)  0.03 (0.01, 0.04) | 425  408  600  600  600 | 1.00  1.00  1.00  1.00  1.01 |
|  | Invertebrate biomass  Low flow  Macrophyte cover | | Adult  Subadult  Adult  Juvenile | 0.03 (0.01, 0.05)  -0.03 (-0.04, -0.01)  -0.04 (-0.07, -0.02)  -0.02 (-0.02, -0.01) | 909  612  600  600 | 1.01  1.01  1.00  1.00 |
|  | **Mean temperature (S-A)** | | Juvenile | 0.04 (0.03, 0.05) | 660 | 1.00 |
|  | Trout abundance  Mean temperature (A-W)  Growth parameter ($L_{\infty}$)  Growth parameter ($K$)  Growth parameter ($t_{0}$) | | Subadult  **Adult**  Juvenile  Subadult  Adult  -  -  - | -0.01 (-0.03, 0.00)  **-0.02 (-0.04, 0.00)**  -0.02 (-0.02, -0.01)  0.03 (0.02, 0.04)  0.05 (0.03, 0.08)  348.24 (345.01, 351.47)  0.14 (0.12, 0.15)  -2.37 (-2.71, -2.00) | 500  544  545  600  596  101  66  64 | 1.00  1.00  1.00  1.00  1.00  1.02  1.04  1.04 |
| 5 – full model fit to data excluding any observed lengths of recaptured individuals (*n* = 4,353). Any change in direction or strength of effects compared to the full model fit to all data are shown in italics. | Grayling abundance  High flow | | Juvenile  Subadult  Adult  Juvenile  Subadult | -0.01 (-0.01, 0.00)  -0.04 (-0.05, -0.02)  -0.15 (-0.18, -0.12)  -0.04 (-0.04, -0.03)  0.01 (-0.01, 0.02*)* | 600  468  600  633  723 | 1.01  1.01  1.00  1.01  1.00 |
|  | Invertebrate biomass  *Low flow*  Macrophyte cover | | Adult  Subadult  *Adult*  Juvenile | 0.04 (0.01, 0.06)  -0.04 (-0.05, -0.03)  *0.00 (-0.03, 0.03)*  -0.02 (-0.02, -0.01) | 1083  643  847  865 | 1.02  1.01  1.00  1.00 |
|  | Mean temperature (S-A) | | Juvenile | 0.04 (0.03, 0.04) | 844 | 1.00 |
|  | Trout abundance  Mean temperature (A-W)  Growth parameter ($L_{\infty}$)  Growth parameter ($K$)  Growth parameter ($t_{0}$) | | Subadult  Juvenile  Subadult  Adult  -  -  - | -0.01 (-0.02, 0.00)  -0.02 (-0.02, -0.01)  0.05 (0.04, 0.06)  0.07 (0.04, 0.10)  337.94 (334.28, 341.79)  0.18 (0.16, 0.20)  -1.59 (-1.86, -1.31) | 600  600  600  704  101  55  56 | 1.00  1.00  1.01  1.00  1.10  1.18  1.17 |

**Table S3.** Model comparison summary. Leave-one-out cross validation statistics suggest that the there was no statistical difference between the models compared. Model 1 is the full model and Models 2 to 4 are the candidate full models with the addition of variables last removed during model simplification for each life-stage.

| **Model** | **LOOIC** | **Elpd_loo** | **p_loo** | **Elpd_diff** | **Se_diff** |
| --- | --- | --- | --- | --- | --- |
| 3 | 48955.4 | -24477.7 | 30.6 | 0.0 | 0.0 |
| 4 | 48955.5 | -24477.7 | 30.8 | 0.0 | 3.0 |
| 2 | 48956.3 | -24478.2 | 30.3 | -0.4 | 2.3 |
| 1 | 48957.4 | -24478.7 | 30.3 | -1.0 | 1.9 |

**
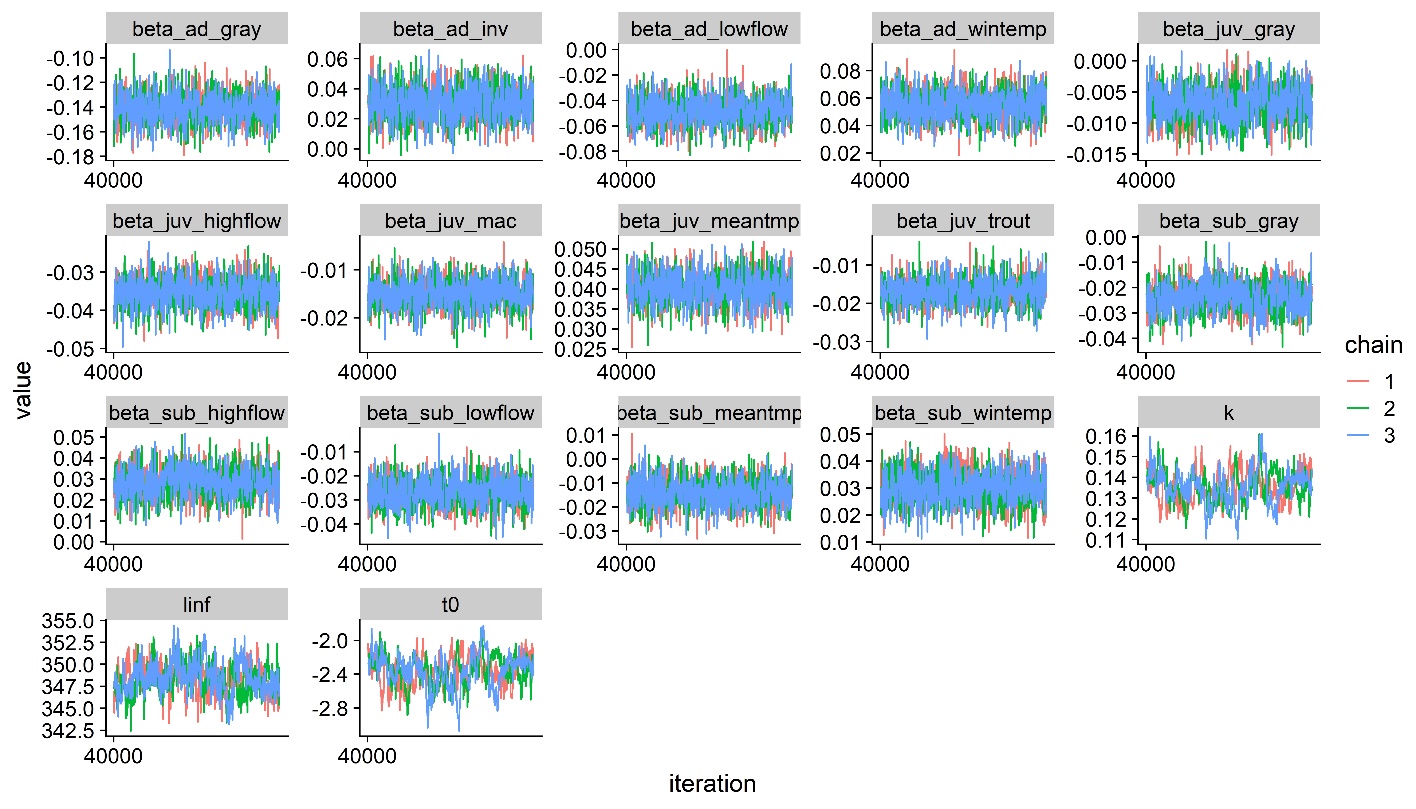
**

**Fig. S6.** MCMC trace plots of estimated model parameters in the full growth model to visually assess convergence of the 3 chains for each estimated model parameter and coefficient.

**Table S4**. Model convergence statistics. Gelman statistic estimates for all estimated parameters in the full growth model. An estimate of 1 suggested converged, stationary and well mixing chains.

| Parameter | Point estimate |
| --- | --- |
| $L_{\infty}$ | 1.01 |
| $K$ | 1.02 |
| $t_{0}$ | 1.02 |
| $\beta_{l,M}$ | All 1.00 – 1.01 |
| $\mu_{y,s,a}$ | All 1.00 – 1.02 |

Code for simulation

# load libraries
library(ggplot2)
library(dclone)
library(data.table)
library(rjags)

## simulation parameter values
linf <- 600 # asymptotic size
k <- 0.2 # Brody growth coefficient
t0 <- -0.5 # age at length 0
nages <-6 # no. of ages (0+ to 5+)
ages <- 1:6 # vector of ages
sd_len <- 15 # sd of length
nyears <- 17 # no. of years (2003 - 2019)
nsites <- 6 # no. of sites (1 - 6)

## coefficient values
beta_effect1 <- 0.25
beta_effect2 <- -0.1
beta_effect3 <- -0.1
effect1 <- rnorm(nyears, 0, 1)
effect2 <- rnorm((nyears*nsites), 0, 1)
effect2_m <- matrix(effect2, nrow = nyears, ncol = nsites)

# simulate length-at-age data --------------------------------------------

# empty array
lena <- array(NA, dim = c(nyears, nsites, nages))

# juvenile (0+) expected length-at-age
for(y in 1:nyears){
 for(s in 1:nsites){

 lena[y, s, 1] <- linf *
 (1-exp(-k * (ages[1] - t0))) *
 exp(beta_effect1 * effect1[y] + beta_effect2 * effect1[y]^2)
 }
}

# sub-adult and adult

# vector of mean lengths for each sub-adult and adult age (1+ to 5+) in year -1
mean_ad_len <- rep(c(350, 420, 550, 580, 590), each = 6) # for simulating
all_len <- rep(c(NA, 350, 420, 550, 580, 590), each = 6) # for jags model

# add mean lengths in year -1 for all ages except juveniles
lena[1, , -1] <- mean_ad_len

# calculate subsequent lengths-at-age in year and site loops
for(y in 1:(nyears-1)){
 for(s in 1:nsites){

 # 1+ expected length-at-age
 lena[y + 1, s, 2] <- lena[y, s, 1] + (linf - lena[y, s, 1]) *
 (1-exp(-k * (ages[2] - t0)))

 # 2+ to 5+ expected length-at-age
 for(i in 3:nages){
 lena[y + 1, s, i] <- lena[y, s, i-1] + (linf - lena[y, s, i-1]) *
 (1-exp(-k * (ages[i] - t0))) *
 exp(beta_effect3 * effect2_m[y + 1, s])
 }
 }
}

## simulate observed data using length-at-age
nobs <- 3400 #(17 year x 200 obs per year) # no. of observations
p_ages <- c(0.509, 0.25, 0.125, 0.067, 0.033, 0.016) # age structure
aobs <- sample(1:nages, nobs, replace = TRUE, prob = p_ages) # obs ages
yobs <- sample(1:nyears, nobs, replace = TRUE) # observed years
sobs <- sample(1:nsites, nobs, replace = TRUE) # observed sites

# empty vector for observed lengths
lobs <- NULL

# calculate observed lengths using lena
for(i in 1:nobs){
 lobs[i] <- rnorm(1,
 lena[yobs[i], sobs[i], aobs[i]],
 sd_len)
}

## plot simulated length at age
la_dat <- data.frame('length' = lobs, 'age' = aobs, 'sites' = sobs, 'year' = yobs)
la_dat$age <- factor(la_dat$age)

la_plot <- ggplot(la_dat, aes(x = age, y = length)) +
 geom_boxplot() +
 facet_wrap(~sites)

print(la_plot)

# model fitting in jags --------------------------------------------------

# model function
f <- function(){

 # juvenile length-at-age
 for(y in 1:nyears){

 for(s in 1:nsites){

 lena[y, s, 1] <- linf *
 (1-exp(-k * (ages[1]-t0))) *
 exp(beta_effect1 * effect1[y] + beta_effect2 * pow(effect1[y], 2))
 }
 }

 # subadult and adult lengths-at-age

 # first year
 for(a in 2:nages){

 mu_lena[1, a] ~ dnorm(0, 0.001)

 for(s in 1:nsites){

 lena[1, s, a] <- mu_lena[1, a]

 }

 }

 # remaining years
 for(y in 1:(nyears-1)){
 for(s in 1:nsites){

 lena[y + 1, s, 2] <- lena[y, s, 1] +
 (linf - lena[y, s, 1]) *
 (1-exp(-k * (ages[2]-t0)))

 for(i in 3:nages){

 lena[y + 1, s, i] <- lena[y, s, i-1] +
 (linf - lena[y, s, i-1]) *
 (1-exp(-k * (ages[i]-t0))) *
 exp(beta_effect3 * effect2[y + 1, s])
 }
 }
 }

 # likelihood
 for(i in 1:nobs){

 lobs[i] ~ dnorm(lena[yobs[i], sobs[i], aobs[i]], tau)
 }

 # priors
 linf ~ dnorm(0, 0.001);T(0,)
 k ~ dgamma(1, 1)
 t0 ~ dnorm(0, 0.001)
 tau ~ dgamma(0.01, 0.01)
 sd_len <- 1/sqrt(tau)
 beta_effect1 ~ dnorm(0, 0.001)
 beta_effect2 ~ dnorm(0, 0.001)
 beta_effect3 ~ dnorm(0, 0.001)

}

## data passing to JAGS
jagdat<-list('nobs'= nrow(la_dat),'lobs'= la_dat$length,'aobs'= la_dat$age, 'nages' = nages, 'ages' = ages, 'effect1' = effect1, 'effect2' = effect2_m, 'yobs' = la_dat$year, 'nyears' = nyears, 'nsites' = nsites, 'sobs' = la_dat$sites, 'ad_len' = matrix(data = all_len, nrow = 6, ncol = 6))

## parameters to monitor
params <- c('linf','k','t0', 'sd_len', 'beta_effect1', 'beta_effect2', 'beta_effect3', 'lena', 'mu_lena')

## mcmc settings
setts <- data.frame('nc' = 3, 'na' = 2000, 'nb' = 2000, 'ni' = 5000, 'nt' = 10)

## setup clusters
cl <- makePSOCKcluster(setts$nc)
parLoadModule(cl, 'lecuyer')
parLoadModule(cl, 'dic')
parLoadModule(cl, 'glm')

## parallel sampling
parJagsModel(cl = cl, name = 'res', file = f,
 data = jagdat, n.chains = setts$nc, n.adapt = setts$na)
parUpdate(cl = cl, object = 'res', n.iter = setts$nb)
s <- parCodaSamples(cl = cl, model = 'res',
 variable.names = params,
 n.iter = setts$ni, thin = setts$nt)

## discard nb iters
foo <- stack(s)

## create df
dt <- data.table(foo, keep.rownames = TRUE)
setkey(dt, variable, chain, iter)

## calculate the quantiles across chains
fit_sum <- dt[, list(rn = rn[1],
 mean = mean(value),
 sd = sd(value),
 min = min(value),
 q2.5 = quantile(value, 0.025),
 q25 = quantile(value, 0.25),
 q50 = quantile(value, 0.50),
 q75 = quantile(value, 0.75),
 q97.5 = quantile(value, 0.975),
 max = max(value)),
 by = list(variable)]
o <- order(as.numeric(fit_sum$rn))
fit_sum <- fit_sum[o, ]
fit_sum$variable <- factor(fit_sum$variable, levels = fit_sum$variable)

## remove variables
fit_sum <- fit_sum[, -'rn']

## stop cluster
parUnloadModule(cl, 'glm')
parUnloadModule(cl, 'dic')
parUnloadModule(cl, 'lecuyer')
stopCluster(cl)

## plot parameter estimates

# subset of par ests
fit_par <- subset(fit_sum, variable %in% c('beta_effect1', 'beta_effect2', 'beta_effect3', 'k', 'linf', 'sd_len', 't0'))

# vector of simulated variable values

sim_var <- sapply(as.character(fit_par$variable), function(v) eval(parse(text = v)))

fit_par$simulated_var <- sim_var

par_est_plot <- ggplot(fit_par, aes(x = variable, y = mean)) +
 geom_point(size = 3, col = 'black') +
 geom_errorbar(aes(ymin = q2.5, ymax = q97.5), width = 0.5, col= 'black') +
 geom_point(aes(x = variable, y = simulated_var), size = 3, col = 'blue') + theme_cowplot() +
 facet_wrap(~variable, scale = 'free') +
 xlab('parameter')

print(par_est_plot)
